# Ultra-fast speech comprehension in blind subjects engages primary visual cortex, fusiform gyrus, and pulvinar – a functional magnetic resonance imaging (fMRI) study

**DOI:** 10.1186/1471-2202-14-74

**Published:** 2013-07-23

**Authors:** Susanne Dietrich, Ingo Hertrich, Hermann Ackermann

**Affiliations:** 1 Center for Neurology/Department of General Neurology, Hertie Institute for Clinical Brain Research, University of Tübingen, Hoppe-Seyler-Str. 3, D-72076, Tübingen, Germany

**Keywords:** Speech perception, Compressed speech, Late- and early-blind subjects, Cross-modal plasticity, Timing

## Abstract

**Background:**

Individuals suffering from vision loss of a peripheral origin may learn to understand spoken language at a rate of up to about 22 syllables (syl) per second - exceeding by far the maximum performance level of normal-sighted listeners (ca. 8 syl/s). To further elucidate the brain mechanisms underlying this extraordinary skill, functional magnetic resonance imaging (fMRI) was performed in blind subjects of varying ultra-fast speech comprehension capabilities and sighted individuals while listening to sentence utterances of a moderately fast (8 syl/s) or ultra-fast (16 syl/s) syllabic rate.

**Results:**

Besides left inferior frontal gyrus (IFG), bilateral posterior superior temporal sulcus (pSTS) and left supplementary motor area (SMA), blind people highly proficient in ultra-fast speech perception showed significant hemodynamic activation of right-hemispheric primary visual cortex (V1), contralateral fusiform gyrus (FG), and bilateral pulvinar (Pv).

**Conclusions:**

Presumably, FG supports the left-hemispheric perisylvian “language network”, i.e., IFG and superior temporal lobe, during the (segmental) sequencing of verbal utterances whereas the collaboration of bilateral pulvinar, right auditory cortex, and ipsilateral V1 implements a signal-driven timing mechanism related to syllabic (suprasegmental) modulation of the speech signal. These data structures, conveyed via left SMA to the perisylvian “language zones”, might facilitate – under time-critical conditions – the consolidation of linguistic information at the level of verbal working memory.

## Background

So far, a variety of studies demonstrated superior auditory-perceptual abilities in blind individuals as compared to sighted controls [1], e.g., enhanced speech discrimination in a noisy environment [2], faster processing of simple sounds like tones [2,3], sharper tuning of spatial attention towards noise bursts [4], higher recognition accuracy of the direction of pitch changes [5] and, finally, improved identification of voices as well as enlarged memory for vocal signatures [6]. Furthermore, functional imaging studies indicate the central-visual system to contribute to the enhanced processing of non-visual stimuli in blind subjects. For example, striate cortex shows significant hemodynamic activation during Braille reading (e.g., [7-11]), auditory motion detection [12], syntactic and semantic speech processing [13] as well as cognitive language tasks such as verb generation, production of mental images based upon animal names, and retrieval of verbal-episodic memory contents [14-16]. Given their superior acoustic-perceptual abilities, the enhanced speech/language skills of blind individuals might be based upon “stimulus-driven” central-auditory mechanisms, operating across linguistic and non-linguistic domains, rather than supramodal “bottom-up” processes. However, activation of visual cortex in blind individuals concomitant with performance benefits has also been observed during verbal tasks in the absence of any sensory input [14]. Such findings cannot easily be explained by, e.g., enhanced temporal resolution of acoustic signals.

As a further “feat” within the realm of acoustic communication, analogous, conceivably, to the fast-reading capabilities of sighted individuals, repeated exposure to accelerated verbal utterances may enable blind people to understand spoken language at speaking rates of up to 22 syllables (syl) per second – an accomplishment exceeding by far the upper limits of untrained subjects (ca. 8 syl/s) [17]. Therefore, patients suffering from vision impairments may considerably benefit, e.g., during academic education, from screen-reading text-to-speech systems, operating at ultra-fast syllable rates [18]. Using functional magnetic resonance imaging (fMRI), a preceding single-case study of our group first documented significant hemodynamic activation of right-hemispheric primary visual cortex (V1) and contralateral fusiform gyrus (FG) in a blind university student with high ultra-fast speech perception capabilities during application of compressed verbal utterances (16 syl/s) whereas, by contrast, similar responses did not emerge in a series of sighted control subjects [19].

As an extension of the previous single-case study, this subsequent investigation tries to confirm the association of ultra-fast speech perception with hemodynamic responses of right V1/left FG at the group-level and to provide first evidence for a specific causal engagement of those structures in enhanced spoken language comprehension. More specifically, it must be expected that hemodynamic activation of right V1/left FG covaries with behavioral measures of ultra-fast speech comprehension capabilities. In order to test this hypothesis, blind subjects varying in their capabilities to understand ultra-fast speech – and sighted individuals never exposed to accelerated spoken language – underwent fMRI measurements while listening to sentence utterances of a moderately (8 syl/s) or ultra-fast (16 syl/s) speaking rate. In addition, the same test materials were applied as time-reversed speech signals (backward played sentences) to the participants – a procedure rendering verbal utterances unintelligible. Those spectrally matched, but “phonologically incorrect” acoustic stimuli served as control items to the two forward-conditions of the experiment.

Functional reorganization of visual cortex – and its impact on an individual’s perceptual, attentional, and cognitive skills – appears to be critically constrained by the time of vision loss (see [20] for a recent review). Nevertheless, both subjects with early- as well as late-onset blindness have been found capable – though, eventually, to varying degrees - to acquire the capacity of ultra-fast speech comprehension [17]. Against this background, the present study recruited a group of late-blind subjects varying in the onset of vision loss as well as three early-blind individuals, allowing for a preliminary, i.e., descriptive analysis of age effects.

## Methods

### Subjects

A total of 14 blind (11 males; mean age = 35.1 years, *SD* = 10.1) and 12 sighted subjects (9 males; mean age = 30.3 years, *SD* = 8.4) participated in the functional imaging experiment (Table 1). All of them were right-handed (Edinburgh handedness inventory) native German speakers without a history of neurological problems or hearing deficits as determined by means of an audiogram. The study design had been approved by the ethics committee of the University of Tübingen, and written informed consent could be obtained prior to the fMRI measurements from all subjects. As a prerequisite to informed consent, all blind participants received the relevant data on, e.g., the experimental procedure, the publication policy etc. in a written format as pdf-files by e-mail. Thus, they could read the files at home, using their text-to-speech systems. Prior to the fMRI measurements, the experimental operator read, in addition, the information materials to each blind individual who was then asked to sign the consent form in face of a sighted witness, i.e., an accompanying person. Since the blind participants had been recruited from community organizations, a detailed clinical data bank was not available to the authors and, thus, information on etiology and follow-up of the ophthalmological disorders mainly had to be drawn from personal interviews and medical reports. In all instances, a peripheral origin of blindness could be established, but the participants represented a rather heterogeneous group with respect to age of onset of vision loss or their residual – in all cases minor – visual capabilities such as light and color sensitivity (see Table 1). A further separation of the participants into subgroups of congenital, early-, or late-onset blindness (see, e.g., [21]) yielded sample sizes too small for any meaningful statistical comparisons (vision loss from, by and large, the date of birth onwards, n = 3; rather abrupt onset between one and 14 years of age, n = 3; blindness emerging after that period, n = 8). Therefore, age at onset of vision loss and disease duration served as a covariate of data analysis.

**Table 1:** **Clinical and behavioral data of the vision**-**impaired and healthy subjects**

**Subjects**	**Speech perception**		**Onset of blindness**	**Etiology of blindness**	**Characterization of visual deficits**
	**Ultra-fast utterances**	**Moderately fast utterances**			
N01	93	94	7	retinal detachment	no residual visual perception, visus 0 bilaterally
N02	91	85	14	retinitis pigmentosa	no statement
N03	78	90	Birth	optic atrophy	no residual visual perception, visus 0 bilaterally
N04	72	80	26	macular degeneration	light perception extant, visus 0.01 bilaterally
N05	67	85	7	hereditary vision loss (gene mutation)	no residual visual perception, visus 0 bilaterally
N06	65	89	44	uveitis intermedia	visus 0.3 bilaterally
N07	64	89	37	glaucoma	no statement
N08	62	82	24	retinitis pigmentosa	light perception extant
N09	60	84	17	retinal damages	visus 0 bilaterally
N10	57	99	13	retinal detachment	no residual visual perception, visus 0 bilaterally
N11	55	100	Birth	optic atrophy	light perception, dim object recognition
N12	39	74	18	retinal damages	visus 0 bilaterally
N13	32	63	Birth	viral infection	no residual visual perception, visus 0 bilaterally
N14	0	65	47	eye cataract, glaucoma	close vision > 24.5 diopter, visus 0 left, 1.5 right
N15	16	85	-	control	
N16	16	99	-	control	
N17	11	69	-	control	
N18	6	61	-	control	
N19	5	89	-	control	
N20	4	71	-	control	
N21	4	67	-	control	
N22	9	81	-	control	
N23	7	85	-	control	
N24	16	96	-	control	
N25	6	88	-	control	
N26	8	78	-	control	

### Stimuli

The test materials of this investigation – a subset of the stimulus corpus of the preceding single-case study [19] – encompassed 40 different text passages (sentences) obtained from newspapers and transformed into acoustic speech signals by means of a formant synthesizer (text-to-speech system) at the Institute of Phonetics of the Saarland University (screen reader software JAWS 2008; male voice; http://www.freedomsci.de). All utterances were first recorded at a normal speaking rate, amounting to 4–6 syl/s. Using the speech processing software Praat (version 4.5; http://www.fon.hum.uva.nl/praat/), 20 out of the total of 40 sentences were compressed to a moderately fast (8 syl/s) and the remaining 20 items to an ultra-fast syllabic rate (16 syl/s; see Additional files 1 and 2). In addition, both subsets of the test materials were stored as time-reversed speech signals (backward played sentences), serving as spectrally matched, but unintelligible and “phonologically incorrect” control items to the two forward-conditions (see Additional files 3 and 4). Duration of the various stimuli extended from ca. 3.5 to 4 s (moderately fast spoken sentences: mean length = 3.84 s, *SD* = 0.15 s; ultra-fast spoken sentences: mean = 3.78 s, *SD* = 0.12 s). Thus, the moderately fast tokens comprised 30.75 syllables per sentence, whereas the ultra-fast stimuli consisted on average of 60.55 syllables per utterance.

### Behavioral data acquisition and analyses

To obtain a quantitative behavioral measure of an individual’s capability to understand moderately fast and ultra-fast speech utterances, each subject performed – outside the scanner and prior to the fMRI measurements – a sentence repetition task, encompassing a subset of the forward stimulus materials (ten sentences at both speaking rates each). These verbal utterances were truncated to the phrase-initial 9–10 words in order to limit memory load. In some instances, this approach yielded incomplete sentences, but those items, nevertheless, represented meaningful “stretches” of spoken language. The stimulus materials (see Additional file 5 for an example) were displayed to the participants via loudspeakers (Fostex, Personal monitor, 6301B) within a sound-attenuated room, subjects being asked to repeat them “as faithful as possible”, even in case not all the words had been “grasped”. The subjects’ repetitions were recorded on digital discs (M-audio Microtrack 2496). Subsequent evaluation included the computation of the percentage of correctly reproduced words at each rate condition, focusing upon word form irrespective of minor grammatical errors such as deviant singular or plural endings. Analysis of the behavioral data included comparison of the speech comprehension capabilities of blind and sighted subjects (two-tailed two-sample *t*-test) as well as correlation analyses within the blind group (two-tailed Pearson tests), addressing the relationship between repetition performance, on the one hand, and age at the onset of vision loss as well as disease duration at the time of fMRI measurements, on the other.

### fMRI – data acquisition

All functional imaging sessions included two repetitions each of the 20 moderately fast (mf), 20 ultra-fast (uf), 20 time-reversed moderately fast (rev-mf), and 20 time-reversed ultra-fast (rev-uf) utterances (= altogether 160 stimuli) as well as 40 silent baseline intervals (scanner noise). The test materials – distributed across five runs – were presented in randomized order (event-related design) at an inter-stimulus interval of 9.6 s (jitter = ± 1.4 s, steps of 0.2 s) via headphones adapted to MRI measurements by removal of their permanent magnets (Sennheiser HD 570, binaural stimulus application). Since these headphones show sufficient dampening of environmental noise, it was not necessary to provide the subjects with earplugs during the experiment. Prior to scanning, participants were instructed to listen carefully to the applied auditory stimuli and to try to understand the displayed verbal utterances. Thus, the design did not allow for an explicit control of speech comprehension during the fMRI experiment. However, the brain structures sensitive to speech intelligibility have been found to “light up” even under listening-only conditions [22]. Activation of language processing areas such as the inferior frontal gyrus (IFG) can be considered, thus, an indicator of actual speech comprehension [23]. Subjects were asked to close their eyes during scanning and to report to the experimenters whether they could adequately hear the test materials in the presence of scanner noise, otherwise the sound amplitude of the stimuli was adjusted.

The experiment was run on a 3 Tesla MRI system (Magnetom TRIO; Siemens, Erlangen, Germany), using an echo-planar imaging sequence (echo-time = 30 ms, 64 × 64 matrix with a resolution of 3 × 3 mm^2^, 27 axial slices across the whole brain volume, TR = 1.6 s, slice thickness = 4 mm, flip angle = 90°, 270 scans per run). The scanner generated a constant background noise throughout fMRI measurements, serving as the baseline condition of the experimental design (null event). Anatomical images required for the localization of the hemodynamic responses were obtained by means of a GRAPPA sequence (T1-weighted images, TR = 2.3 s, TE = 2.92 ms, flip angle = 8°, slice thickness = 1 mm, resolution = 1 × 1 mm^2^) of a bi-commissural (AC-PC) orientation.

### FMRI – data analyses

Preprocessing of the data encompassed slice time and motion correction, normalization to the Montreal Neurological Institute (MNI) template space, and smoothing by means of an 8 mm full-width half-maximum Gaussian kernel (SPM5 software package; http://www.fil.ion.ucl.ac.uk/spm). For the sake of statistical analysis, the blood oxygen level-dependent (BOLD) responses were modeled by means of a prototypical hemodynamic function within the context of a general linear model (event durations = 4 s). Any low-frequency temporal drifts were removed using a 128 s high-pass filter.

The evaluation of the functional imaging data encompassed the following steps of signal analysis:

a) In order to delineate the brain regions engaged in the processing of the various stimulus categories considered (mf, uf, rev-mf, rev-uf), the contrast of hemodynamic activation versus baseline was computed separately for blind and sighted individuals (whole-head one-sample *T*-test, threshold at voxel level = *p* < .001 uncorrected, threshold at cluster level = *p* < .05 corrected; the Additional file 6 includes the respective SPM coordinates). Evaluation of the differences between the blind and sighted groups under the various conditions (mf, uf, rev-mf, rev-uf, versus baseline each) was based upon a whole-head two-sample *T*-test (threshold at voxel level = *p* < .001 uncorrected, at cluster level = *p* < .05 corrected; the respective results can be found in the Additional files 7 and 8).

b) A whole-head covariance analysis was conducted, allowing for the identification of hemodynamic responses correlated with ultra-fast speech comprehension capabilities as measured outside the scanner (ultra-fast speech versus baseline condition, pooled across blind and sighted participants). The whole-head random-effects model for group analyses was corrected for multiple comparisons across the entire brain (threshold at voxel level = *p* < .0005 uncorrected, threshold at cluster level = *p* < .05 corrected). In order to characterize the lateralization effects of the BOLD responses within V1 bound to the SPM *T*-contrast “ultra-fast versus baseline”, the signal changes (in percent) confined to a respective anatomical mask were calculated separately for the left and right hemisphere, using a repeated measures ANOVA (early- and late-blind individuals) with the intra-subject factor *Hemisphere* (left/right) and the between-subject factor *Group* (late-/early-blind).

c) The data from late- and early-blind subjects were analyzed separately to detect activation differences between those subgroups within brain regions contributing to ultra-fast speech processing, based upon the SPM between-group *T*-contrast “blind versus sighted” (all conditions pooled versus baseline; see Additional file 9). In addition, the late-blind group – excluding the three early-onset subjects – underwent a covariance analysis (SPM *T*-contrast “ultra-fast versus baseline”; see Additional files 10 and 11).

d) In order to resolve the confounding effects between blindness and ultra-fast speech perception within the initial covariance test, post hoc regions-of-interest (ROI) analyses were performed, testing the relation of behavioral performance and hemodynamic responses separately for the blind and sighted subgroups, considering both moderately fast and ultra-fast speech conditions (versus baseline). Because of a strong ceiling effect, behavioral performance – in terms of the understanding of moderately fast speech – was not taken into account here. Under these conditions, individual variation might reflect differences in memory load or the impact of attentional factors, rather than specific mechanisms engaged in ultra-fast speech comprehension.

The selected ROIs represent the significant activation clusters provided by the preceding whole-head covariance analyses (across all subjects: early-blind, late-blind, and sighted). The following ROIs were considered for analysis: (i) right V1, (ii) left FG, (iii) left IFG, (iv-vi) three central-auditory regions adjacent to Heschl’s gyrus - approximately corresponding to the anterior part of left-hemispheric superior temporal sulcus (aSTS) and to the posterior compartment of the superior temporal sulcus (pSTS) of either hemisphere, (vii) left supplementary motor area (SMA), (viii) left precentral gyrus (PrCG), and (xi-x) bilateral pulvinar (Pv). The ROI spheres (radius = 4 mm) centered around the peak coordinates as derived from the preceding SPM covariance analysis (see Table 2).

**Table 2:** **Coordinates of the whole**-**head covariance analysis**

**Anatomical region**	**Side**	**Cluster size (voxel)**	**MNI coordinates**			**T value**
			**x**	**y**	**z**	
Cuneus, BA17 / 18	Right	368	15	-102	6	7.61
Cerebellum Crus2	Right		21	-78	-39	6.11
Inferior frontal gyrus, pars triangularis, BA44 / 45	Left	162	-48	18	18	6.15
Fusiform gyrus	Left	159	-42	-51	-21	6.07
Supplementary motor area	Left	112	-6	9	60	5.42
Posterior middle temporal gyrus / superior temporal sulcus	Left	216	-57	-48	6	5.20
Anterior middle temporal gyrus / superior temporal sulcus	Left	54	-51	-12	-15	5.18
Posterior middle temporal gyrus / superior temporal sulcus	Right	49	57	-36	6	4.84
Precentral gyrus	Left	51	-42	0	36	4.60
Pulvinar	Left	35	-18	-30	3	4.69
Pulvinar	Right	23	18	-30	6	5.45

Hemodynamic effects of ultra-fast speech comprehension capabilities as a covariate of the SPM *T*-contrast (ultra-fast versus baseline). Responses exceeding a threshold of *p *< .0005 (uncorrected) at a voxel level and a threshold of *p*< .05 (corrected) at a cluster level, comprising an extent threshold of *k *= 46 (contiguous voxels), are included – in addition, hemodynamic activation of left- and right-hemispheric pulvinar regions, though non-significant at the level of the corrected threshold of *k* = 46. Abbreviations: BA, Brodman area; MNI, Montreal Neuroscience Institute template; T, height threshold.

In order to further delineate within three exemplary ROIs, i.e., right V1, left FG, left IFG, the impact of the various experimental factors, i.e., *Group* (blind/sighted), *Meaningfulness* (forward/backward), and *Speaking rate* (ultra-fast/moderately fast), upon the BOLD responses, repeated measures ANOVAs were conducted (see Additional file 12). Determination of the relationship between the capability of ultra-fast speech comprehension, age at blindness onset, duration of vision loss, on the one hand, and the strength of hemodynamic responses (percent BOLD signal changes), on the other, relied upon correlation analyses of the data obtained from the group of blind subjects (two-tailed Pearson tests, significance threshold set at *p* < .05; see Additional file 13).

## Results

### Behavioral data

Comprehension of ultra-fast speech utterances (16 syl/s) – in terms of the percentage of correctly reproduced items of 10-word sentences outside the scanner – extended in blind listeners from 0 to 93% (mean = 59.7%, *SD* ± 24.0%) – a wide range of performance allowing for subsequent correlation analyses. In sighted individuals, performance level consistently fell below 16% (mean = 9.0%, *SD* ± 4.7%) (Table 1). Moderately fast utterances (8 syl/s) yielded comparable results in both subject groups (range across all participants: 61 to 100%; sighted controls: mean = 80.8%, *SD* ± 11.8%; blind listeners: mean = 84.1%, *SD* ± 11.0%). By contrast to the latter condition (two-sample t-test; *T* = 0.74, *p* = .470), blind and sighted individuals significantly differed with respect to speech comprehension of ultra-fast test materials (two-sample t-test; *T* = 7.74, *p* < .001). The enhanced perceptual skills of blind listeners did not show a significant correlation with either the onset of vision loss (*r* = -.347, *p* = .224) or with the duration of blindness at the time of the experiment (*r* = .152, *p* = .604).

### Whole-head fMRI analyses

**Figure 1:**
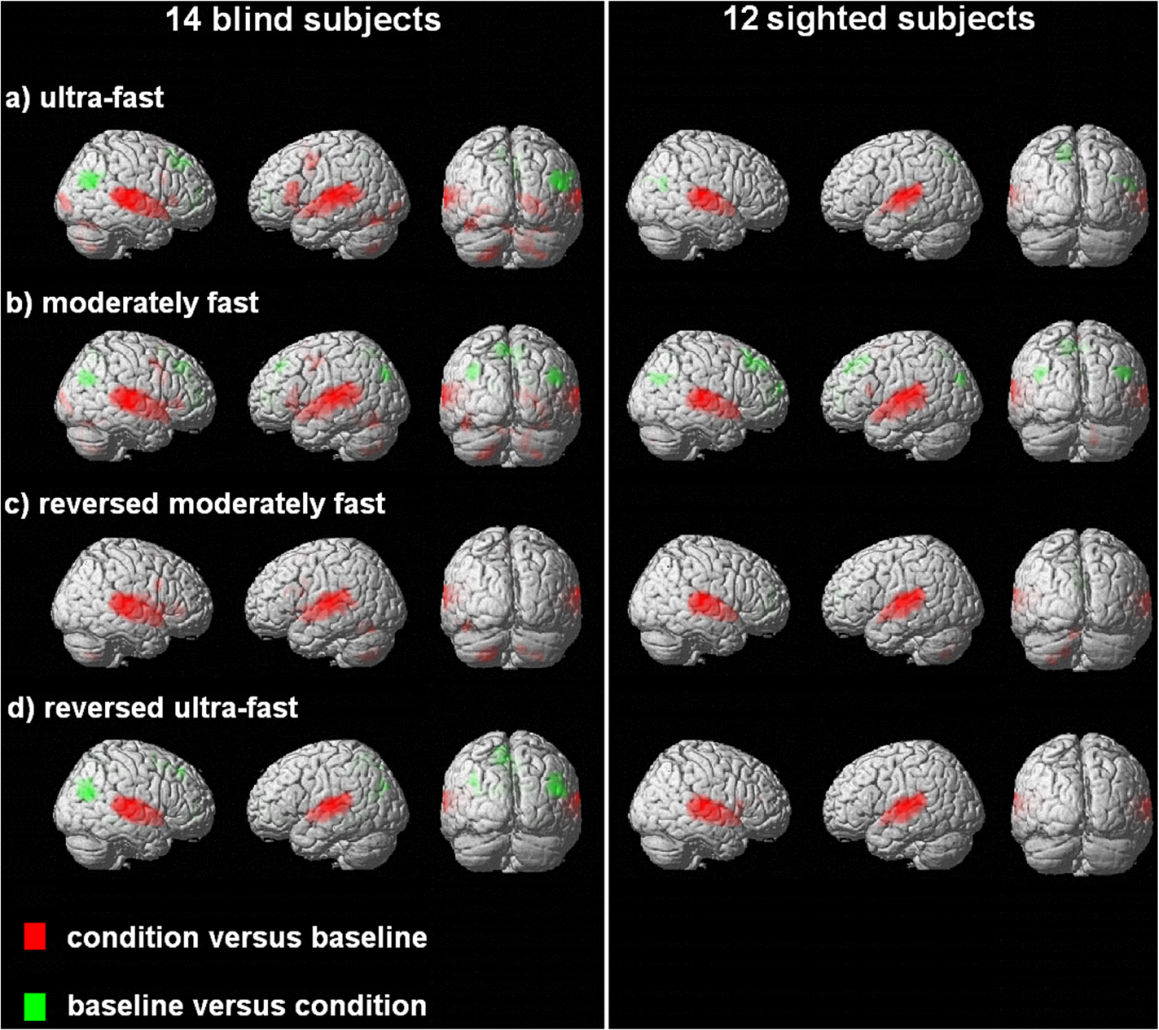
**Whole-head analyses (rate conditions versus baseline each) in the patient and control group. **Hemodynamic responses (SPM *T*-contrasts) to the four experimental conditions (versus baseline and vice versa; a, b: ultra-fast and moderately fast forward speech; c, d: moderately fast and ultra-fast time-reversed speech), displayed separately for the blind and sighted group (activation clusters exceeding a threshold (uncorrected) of *p *< .001 at a voxel level and (corrected) of *p *< .05 at a cluster level). The respective SPM coordinates can be found in the Additional file 6.

All four test materials gave rise to significant BOLD signal changes within primary auditory areas of either hemisphere as well as adjacent structures of the superior temporal cortex both in blind and sighted subjects (SPM *T*-contrasts for each condition versus baseline, conducted separately for the blind and sighted subject groups; see Figure 1 and Additional file 6). In addition, intelligible verbal utterances, i.e., ultra-fast speech in case of skilled blind and moderately fast sentences in case of all participants, elicited significant hemodynamic responses within the superior/middle temporal gyri and sulci, left-hemispheric IFG, left SMA, left PrCG (sighted subjects failed to achieve the threshold of p < .001), as well as the cerebellum. As expected, only the blind participants displayed significant activation of right-hemispheric V1 and left FG during the application of ultra-fast test materials (SPM *T*-contrasts between-group analyses, considering each condition versus baseline; see Additional files 7 and 8). The hemodynamic responses of right V1 and left FG to unreversed moderately fast speech were also restricted to individuals suffering from vision loss (threshold of p < .001 uncorrected at a voxel level; see Additional files 7 and 8). However, activity of visual cortex revealed to be considerably reduced during listening to reversed speech (see Figure 1 and Additional file 6 for descriptive inferences).

**Figure 2:**
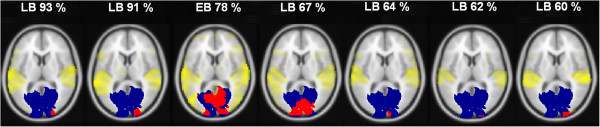
**Individual occipital responses to ultra-fast speech in blind subjects. **BOLD responses during the ultra-fast condition versus baseline (yellow color) obtained from the seven blind participants (1 early-blind = EB; 6 late-blind = LB) with a performance level of ultra-fast speech perception above 60% correctly repeated words. Activated voxels encroaching upon the anatomical mask of the primary visual area (V1 mask: blue color; V1 overlapping activation: red color) were predominantly located within the right hemisphere. Hemodynamic responses exceeding a threshold of *p *< 0.001 (uncorrected) at a voxel level are displayed on horizontal brain sections (x = 0, y = -84, z = 9).

Since the whole-head fMRI analyses of the present study relied upon an anatomical template (V1 mask of SPM software), the observed occipital responses are not necessarily bound to V1 in each participant. Figure 2 exemplifies individual activation spots overlapping the V1 mask in the seven blind subjects (6 late-blind, 1 early-blind patients) with a behavioral performance level above 60% correctly repeated words. All these participants showed activated voxels within V1 during the “ultra-fast versus baseline” condition. Most noteworthy, the right hemisphere comprised significantly stronger hemodynamic responses than the left side (factor *Hemisphere*: *F* (1, 12) = 10.43, *p* < .01), although some participants displayed a more bilateral activation pattern as exemplified in Figure 2 (3rd and 4th display from left) for an early-blind and a late-blind subject.

**Figure 3:**
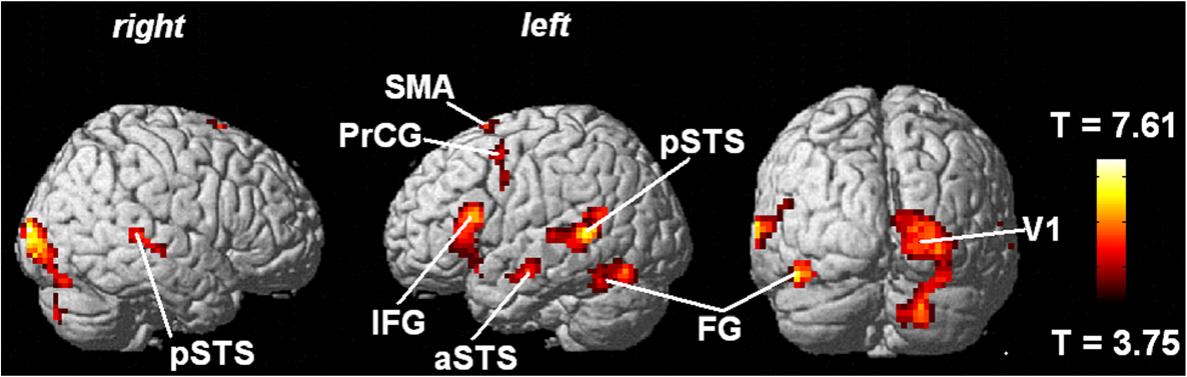
**Whole-head covariance analysis (ultra-fast speech perception as a covariate).** SPM random-effects group analysis (14 blind, 12 sighted subjects): Effects of ultra-fast speech comprehension capabilities (= covariate) within the SPM *T*-contrast “ultra-fast speech versus baseline”. Left- and right-hemispheric hemodynamic responses exceeding a threshold of *p *< 0.0005 (uncorrected) at a voxel level and *p *< 0.05 (corrected) at a cluster level are color-coded in terms of *T *values (height threshold). The respective SPM coordinates are listed in Table 2. Abbreviations: aSTS, anterior superior temporal sulcus; FG, fusiform gyrus; IFG, inferior frontal gyrus; PrCG, precentral gyrus; pSTS, posterior superior temporal sulcus; SMA, supplementary motor area; V1, primary visual area.

When the obtained behavioral measures were entered as covariates into statistical analysis, a significant correlation between the capability of understanding ultra-fast utterances and hemodynamic activation emerged within (i) right-hemispheric V1, including Brodman areas (BA) 17 and 18, (ii) left FG, (iii) left IFG, (iv) the antero-ventral bank of left STS, (v) the postero-ventral bank of STS at either side, extending to the middle temporal gyrus, (vi) left SMA, (vii) left PrCG, and (viii) Pv of both hemispheres (Figure 3, Table 2).

### Differential activation effects in late- and early-blind patients

**Figure 4:**
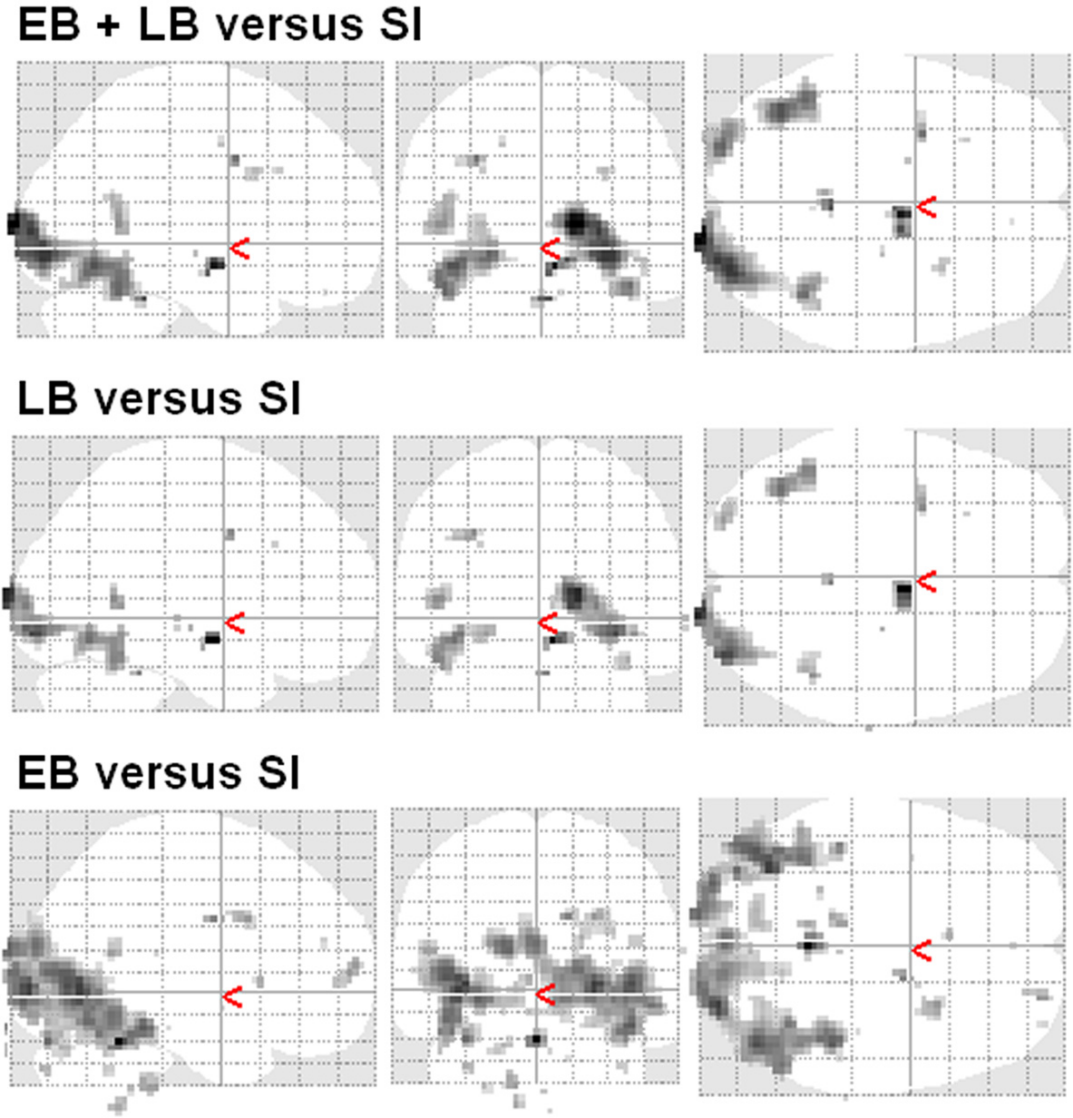
**Whole-head between-group analysis (blind versus sighted). **Displayed are the SPM *T*-contrasts of between-group analyses, (blind versus sighted) based on the condition “all versus baseline” (threshold p < 0.005 (uncorrected) at a voxel level). Upper row: Early- (EB) and late-blind (LB) participants pooled against sighted controls (SI); middle row: late-blind patients versus sighted controls; lower row: early-blind individuals versus sighted controls. The respective SPM coordinates are listed in the Additional file 9.

Whole-head analyses (see above) had revealed right-lateralized hemodynamic activation of V1 concomitant with predominantly left-hemispheric FG responses in blind individuals during ultra-fast speech perception, but not in the sighted control subjects (see also the Additional files 7 and 8). Both the early- (n = 3) and late-blind participants (n = 11) display enhanced BOLD responses of those structures at an uncorrected threshold (voxel level) of *p* < .005 (SPM *T*-contrast “all versus baseline”) – though these occipital responses appear to be characterized by a more extensive and a more bilateral distribution in the three individuals with an early onset of vision loss (descriptive comparison of the SPM between-group *T*-contrasts; see Figure 4 and Additional file 9). The small number of early-blind subjects precludes any direct statistical comparisons with the subgroup of late-blind individuals. Nevertheless, several lines of evidence indicate similar lateralization effects at the level of V1 and FG across all subjects with vision loss. (i) The whole-head covariance analysis (see above), including all participants (early-, late-blind, sighted), had revealed right V1, left FG, temporal, and left IFG activation during ultra-fast speech perception (see Figure 3 and Table 2). Similar results – but at slightly differing significance levels – could be obtained after removal of the early-blind and/or the sighted subjects from analysis (see Additional files 10 and 11). Thus, both early- and late-blind individuals seem to display right-lateralized occipital responses and predominantly left-hemispheric FG activation – associated with the ability to understand ultra-fast speech. (ii) Percent BOLD signal change within the V1 anatomical mask was calculated in order to detect eventual lateralization effects of the hemodynamic responses obtained under the SPM *T*-contrast “ultra-fast versus baseline”. The BOLD responses were found to differ significantly between the left and right hemisphere (see above). Whereas the main effect of the factor *Group* (early-/late-blind) failed statistical significance (*F* (1, 12) = 3.14, *p* = .102), an interaction *Hemisphere* × *Group* emerged at the level of V1 (*F* (1, 12) = 12.60, *p* < .01) – in terms of enhanced responses at the right side in early-blind subjects. However, subsequent post hoc analyses did not yield a significant lateralization effect at the group level (*T* = -1.68, *p* = .218).

### Region-of-interest (ROI) analyses

**Figure 5:**
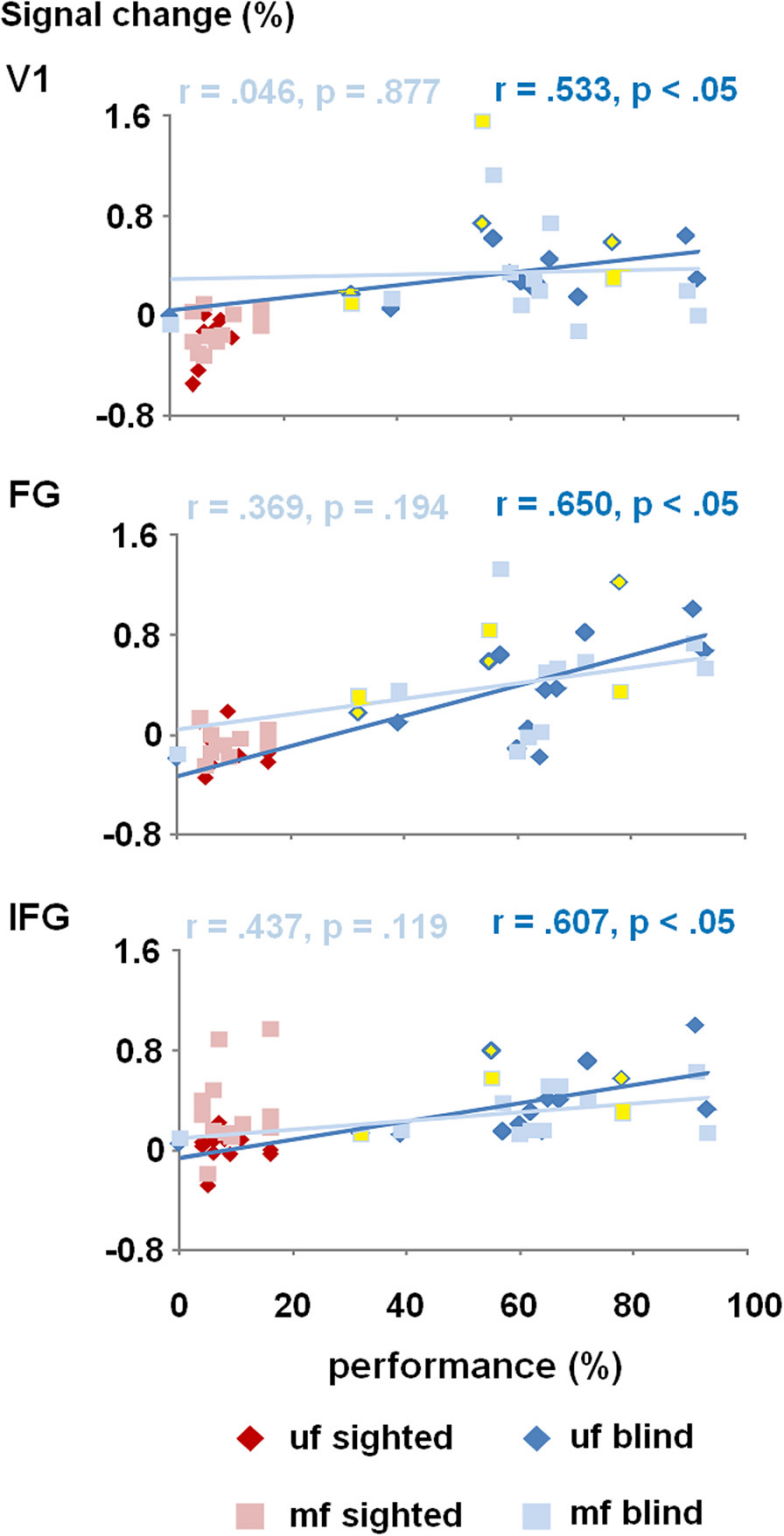
**Region-of-interest (ROI) analyses. **Percent signal change during the ultra-fast and moderately fast listening conditions plotted against individual behavioral performance during application of ultra-fast test materials – as determined prior to scanning – within three selected ROIs, i.e., right-hemispheric primary visual cortex (V1), left-hemispheric fusiform gyrus (FG), and ipsilateral inferior frontal gyrus (IFG). (Note, because of “ceiling effects”, the behavioral data bound to moderately fast utterances have not been included). Regression lines, correlation coefficients, and significance levels refer to the performance of the blind group during ultra-fast (dark blue lines or values, respectively) and moderately fast conditions (light blue; for further data see Additional file 13). As concerns the blind group, the three early-blind subjects are indicated by an extra-label (yellow values).

The significant clusters of hemodynamic activation – as determined by the preceding whole-head covariance analyses – served as a basis for the determination of ROIs. As an example, Figure 5 displays the BOLD signal changes during the ultra-fast and moderately fast condition (versus baseline) within three of the altogether ten ROIs considered – plotted against the percentage of correctly reproduced words of the ultra-fast test materials. As concerns the group of blind subjects, all ROIs showed a significant positive trend towards stronger hemodynamic activation under the ultra-fast speech condition in case of enhanced ultra-fast speech perception capabilities (V1: *r* = .533, *p* < .05; FG: *r* = .650, *p* < .05; IFG: *r* = .607; *p* < .05; Additional file 13 provides the complete set of statistical data, including all ROIs). By contrast, the sighted group did not show any significant correlations between the hemodynamic responses obtained under the two speech conditions and ultra-fast speech comprehension capabilities (see Additional file 13). Furthermore, no significant relationships between the BOLD responses within the various ROIs and, first, age of blindness onset (e.g., V1: *r* = -.298, *p* = .301; FG: *r* = -.243, *p* = .403) and, second, duration of blindness at the time of fMRI measurements could be noted (V1: *r* = -.225, *p* = .439; FG: *r* = .413, *p* = .142; see Additional file 13 for further data). Finally, hemodynamic responses to moderately fast verbal utterances did not show any significant correlations with ultra-fast speech comprehension capabilities in blind listeners (see Additional file 13), apart from left aSTS (*r* = .586, *p* < .05), left Pv (*r* = .688, *p* < .01), and right Pv (*r* = .720, *p* < .01).

As concerns the impact of the various experimental factors upon the BOLD responses within right V1 and left FG (see Additional file 12), the group of blind individuals showed stronger responses to forward as compared to reversed speech (interaction *Group* × *Meaningfulness*, FG (1, 24) = 22.10, *p* < .001), right V1 (*F* (1, 24) = 12.63, *p* < .002). Furthermore, a significant main effect of *Group* emerged within V1 and FG (see Additional file 12) in terms of significant positive mean values in response to forward speech (mf: *T* = 2.73, *p* < .017; uf: *T* = 5.52, *p* > .000) and reversed moderately fast speech (*T* = 2.77, *p* < .016; rev-uf: *T* = 1.90, *p* = .079). By contrast, significantly negative values arose within the sighted group during both forward conditions (one-sample *T*-test; mf: *T* = -2.61, *p* < .024; uf: *T* = -2.65, *p* > .023). Finally, a significant three-way interaction *Speaking rate* × *Group* × *Meaningfulness* (see Additional file 12) could be observed at the level of left-hemispheric IFG (*F* (1, 24) = 18.51, *p* < .000) indicating this region to be sensitive to intelligibility, based on the skill of blind listeners to understand ultra-fast forward speech (post hoc analysis, blind > sighted (uf), IFG: *T* = 4.08, *p* < .001). By contrast, sighted controls showed significant hemodynamic activation of these areas only during the moderately fast forward speech.

## Discussion

### Summary of results

Various left-hemispheric perisylvian structures known to support the perception of spoken language showed, as expected, significant hemodynamic responses to the test materials of this study both in sighted and blind subjects. Furthermore, the precentral gyrus of the same side and the cerebellum displayed significant BOLD signal changes under both forward speech conditions as well as reversed moderately fast speech. These observations are in line with clinical and functional imaging data pointing at a contribution of those structures – under specific circumstances – to auditory speech perception. For example, the cerebellum has been found to engage in the encoding of specific temporal-linguistic information during word identification tasks [24].

Similar to a preceding single-case study [19], individuals with vision loss exhibited significant hemodynamic activation both of right-hemispheric V1 and contralateral FG – covarying with capabilities of ultra-fast spoken language comprehension. More specifically, the BOLD signal changes within these two areas showed a positive correlation with individual ultra-fast speech perception skills and, obviously, depended upon semantic, syntactic, and/or phonological content since time-reversed (backward) speech stimuli were associated with reduced hemodynamic activation. Visual inspection of the data, furthermore, suggests a more extensive and a more bilateral occipital response pattern in the three early-blind as compared to the late-blind individuals. In addition, a positive correlation between BOLD signal magnitude and the level of ultra-fast speech understanding emerged at the level of Pv on either side.

### Interactions between left FG and left perisylvian cortex in blind listeners during ultra-fast speech perception

FG is embedded into the so-called ventral route of the central-visual system which, especially, engages in object recognition (e.g., [25]), but may contribute to phonological operations as well (e.g., [26]). Repeatedly, first, functional imaging studies found this region to support pre-lexical stages of reading tasks and, more specifically, to “house” visual word forms [27]. Among other things, FG has been observed to respond to spoken lexical items even in sighted people (e.g., [28]). Second, impaired speech sound processing in children with reading difficulties seems to be associated with diminished connectivity between FG and frontal language areas [29]. Conceivably, thus, left FG cooperates with the posterior and anterior perisylvian “language zones” – more specifically, ipsilateral IFG and aSTS as well as bilateral pSTS – during ultra-fast speech comprehension. Clinical and functional imaging data indicate a contribution of left IFG to spoken language perception – at least in case more demanding segmentation processes and/or working memory operations are involved [30]. Left pSTS has been found to respond to acoustic signals conveying phonetic-phonological information, irrespective of intelligibility, whereas hemodynamic activation of the anterior part of the same sulcus is restricted to meaningful verbal stimuli [22,31]. Whereas both phonemic as well as non-phonemic sound structures elicit BOLD signal changes within bilateral pSTG, responses of left-hemispheric anterior and middle STS are restricted to familiar consonant-vowel syllables [32]. Furthermore, pSTS at either side has been found associated with phonological aspects of speech recognition [33]. Against this background, the observed temporal lobe activation pattern might be associated with the conveyance of information into higher-order supramodal cortical structures such as (i) the left temporo-parieto-occipital junction, supporting meaning-based representations (auditory-to-meaning interface), and (ii) left-hemispheric frontal areas, providing access to speech production units and phonological working memory (auditory-motor interface) (see [34] for a review). Presumably, left FG representing a secondary phonological area expands the phonological network to cope with the higher processing demands during ultra-fast speech perception.

### Lateralized, i.e., predominantly right-hemispheric hemodynamic activation of V1 in blind listeners during ultra-fast speech perception

The present investigation supports the suggestion that, indeed, visual cortex contributes in a causal manner to enhanced auditory speech processing skills in blind subjects since, first, the capability of ultra-fast spoken language comprehension covaried with the strength of hemodynamic activation of right V1 and, second, the involvement of this structure was considerably reduced during listening to reversed, i.e., non-meaningful test materials.

A series of studies indicate age of blindness onset to significantly constrain the capacity for structural/functional reorganization of central-visual areas in humans. More specifically, only individuals suffering from congenital blindness – lacking any stimulus-driven elaboration of the visual system – appear to be able to “mold” occipital cortex in a fundamentally different manner as compared to sighted subjects [35]. It is, furthermore, still a controversial issue in how far late-onset visual deficits may induce cortical reorganization in terms of functional cross-modal plasticity. For example, neuroimaging studies point at a decline of those capabilities after an age of 14–16 years [21,36]. And Wan and colleagues [37] found early, but not late (≥ 14 years) vision loss to enhance auditory perception during non-speech tasks. The present investigation did not find any significant correlations between the time of onset or the duration of blindness, on the one hand, and the ability to understand ultra-fast speech as well as hemodynamic activation of visual cortex, on the other. Thus, the extent of the recruitment of the central-visual system appears primarily to correlate with behavioral performance rather than the age at vision loss (see [38,39] for similar data). Nevertheless, a significant impact of this clinical parameter upon cross-modal fMRI effects cannot securely be excluded since both high-performing (performance > 60% correctly repeated words) early-blind participants, but only a single skilled late-blind individual (1 out of 5 subjects) displayed bilateral occipital responses. By contrast, a right-lateralized distribution emerged in most late-blind individuals. Similarly, Braille reading was reported to induce responses of the visual cortex at either side in early-blind subjects, whereas late-blind individuals display an activation pattern restricted to the hemisphere ipsilateral to the reading hand [40]. Although the rather small and heterogeneous sample of blind subjects of the present study precludes any firm conclusions, ultra-fast speech perception does not appear to depend upon major rewiring of visual cortex – comparable to the reorganizational processes bound to congenital blindness. Rather, this perceptual capability seems associated with task-dependent cross-modal functional plasticity based, conceivably, on the engagement of existing anatomical structures.

Principally, recruitment of – predominantly right-hemispheric – occipital cortex during ultra-fast speech comprehension could either reflect early, i.e., signal-related computational operations or could be bound to higher-order processing stages, succeeding semantic speech encoding. Previous studies found speech- or language-related tasks such as verbal memory or verb generation tests to yield, as a rule, bilateral hemodynamic activation of occipital cortex – in the presence of more pronounced left-sided responses [14,41]. Hemodynamic activation of primary visual areas at either side also could be documented in blind individuals listening to meaningful as well as meaningless sentences [13]. Again, Braille reading yielded bilateral occipital responses, slightly enhanced within the hemisphere contralateral to the “reading” hand ([8], see also [42]). By contrast, the observed hemodynamic activation of V1 in blind listeners during ultra-fast speech perception displayed strong lateralization effects toward the right side. Thus, the distinct informational cues of the acoustic signal facilitating speech perception under time-critical conditions might be predominantly processed within the non-language-dominant hemisphere. Short spectro-temporal “segments” of the acoustic signal, extending across time intervals of a few tens of milliseconds, encode most of the information related to single speech sound categories such as the various consonants of a language system (e.g., [43]). Important acoustic features within this domain are, e.g., the formant transitions and the voice onset time of stop consonants. It is well established that the extraction of those segmental aspects of spoken language mainly depends upon left-hemispheric perisylvian “language zones”, including anterior and posterior aspects of the superior temporal lobe and posterior ventro-lateral frontal cortex [30,44,45]. Besides those segmental aspects, the acoustic speech signal conveys suprasegmental (prosodic) information such as the intonation of an utterance (“sentence melody”), related to the fundamental frequency contour of the speech signal. In addition, prosodic information also encompasses the specification of temporal structures such as rhythmic and metric patterns [46,47]. By contrast to left-lateralized encoding of the segmental level of verbal utterances, various sources of evidence indicate primarily contralateral representation of suprasegmental/prosodic speech information (e.g., [48]). In case of formant-synthesized verbal utterances, such as the test materials used in the present study, the prosody of spoken language is more or less restricted to syllable timing (syllabic rhythm) as reflected in the speech envelope, i.e., the low-pass-filtered intensity contour of the acoustic signal. A recent whole-head magnetoencephalography (MEG) study, including stimulus materials (ultra-fast and moderately fast speech) similar to the present fMRI investigation, provides additional evidence for a direct translation of the acoustic correlates of syllable structure into electrophysiological brain activity [49]. Most noteworthy, electrophysiological recordings found the speech envelope to be predominantly processed within the right hemisphere [50]. Conceivably, the observed occipital lateralization effects during ultra-fast speech perception in blind subjects indicate V1 to engage in the analysis of the speech envelope or, more specifically, syllabic rhythm. Against this background, activation of the central-visual system might also be expected in case of unintelligible reversed speech. However, Ahissar and colleagues [51] reported a significant correlation between signal-driven syllable-related brain activity of auditory cortex and speech comprehension. This observation could be explained by top-down processes bound to expectations related to the sound structure of the incoming signal which interact with the initial processing of the auditory input. Assuming, thus, right-lateralized early prosodic processing, occipital pole responses correlating with ultra-fast speech comprehension might reflect signal-driven rather than higher-order comprehension processes.

### Mechanisms of ultra-fast speech perception: facilitated verbal consolidation under time-critical conditions

Blind individuals have been found to outperform sighted subjects in tasks requiring temporal order judgments of backward-masked tone stimuli, particularly, in case of brief intervals (40 ms) between the respective auditory events [52]. This condition resembles, by and large, ultra-fast speech since each syllable can be expected to act as a potential masker of the preceding one. Stevens and Weaver [53] assigned the increased temporal resolution of non-speech acoustic events in blind subjects to “perceptual consolidation”, i.e., higher-order processing stages such as auditory working memory, rather than the analysis of spectro-temporal signal characteristics. These suggestions might provide a basis for the explanation of the observed mesiofrontal engagement in the perception of accelerated verbal utterances. Besides visual cortex, hemodynamic activation of left SMA was found to covary with the ability to comprehend ultra-fast spoken language. Several studies indicate this mesiofrontal area to engage in the syllabic organization of verbal utterances during speech production [54-56]. On a broader scale, SMA appears to support timing processes across various sensorimotor and cognitive domains [57,58]. Furthermore, clinical as well as experimental studies point at a contribution of SMA also to speech perception and verbal working memory [59-63]. Since the verbal encoding of longer stretches of speech such as the test materials of the present study must be expected to engage short-term memory processes (see [64]) and since SMA appears to act as a platform of timing operations, related, among other things, to verbal working memory functions, right V1 might provide a “fast track” channel conveying temporal information on syllable structure directly from primary auditory areas via left SMA into verbal working memory. More specifically, the cooperation of primary auditory areas, right V1, and left SMA could facilitate a signal-driven timing mechanism for the transformation of the acoustic signal into a stable (consolidated) verbal code under time-critical conditions.

### The role of the pulvinar during ultra-fast speech perception: synchronization of central-visual and –auditory areas

Besides several cortical regions, ultra-fast speech comprehension capabilities also covaried with the hemodynamic responses of Pv at either side. Animal data obtained in tree shrews indicate those thalamic nuclei to project to V1 as well – in addition to higher-order areas of the central-visual system [65]. As concerns primates, at least some Pv subcomponents are embedded into reciprocal connections with both striate and extrastriate areas (e.g., [66]). In consideration of this network architecture, the respective parts of the Pv have been assumed to support attentional processes operating within the visual domain. Furthermore, tract-tracing studies in monkeys found both the ascending auditory pathways as well as the optic tracts to send convergent collateral fiber tracts to deep layers of the superior colliculus, and the respective target neurons, in turn, project via Pv to auditory as well as visual cortex [67]. Among other things, the pulvinar contributes to the detection of temporo-spatial coincidences of audiovisual signal configurations [68]. In blind subjects, Pv might help to synchronize – driven by acoustic input – striate cortex with the central-auditory system during ultra-fast speech perception, based upon cross-modal subcortical pathways that in sighted individuals subserve audiovisual coincidence detection and the control of visual attention. Given, furthermore, direct anatomical connections between auditory and visual areas [69-71], early multisensory convergence processes at the cortical level must be assumed – as demonstrated, e.g., by means of transcranial magnetic stimulation [72]. These considerations suggest the observed hemodynamic responses within bilateral Pv and primary visual areas, to reflect early (thalamo-cortical) rather than later (cortico-cortical) stages of ultra-fast speech processing.

### Contribution of visual cortex to the perception of time-compressed speech in normal subjects

In principle, speech perception represents an audiovisual process, and under difficult acoustic conditions lip reading may considerably improve spoken language understanding. It must be expected, thus, that the visual system encompasses – to some extent – preconfigurated connections with the auditory system providing a basis for interactions between the two modalities. Indeed, a recent Diffusion Tensor Imaging study (DTI) – evaluating white matter parameters in children – found inter-subject differences in fractional anisotropy to correlate with the comprehension of time-compressed speech [73]: Moderately manipulated signals (40% compression) yielded these effects in white matter areas adjacent to audiovisual association cortex and posterior cingulate gyrus while a greater degree of compression resulted in changes of tracts adjoining prefrontal areas (dorsal and ventral).

A previous fMRI study reported compressed as compared to normal speech to elicit a “convex” distribution pattern of hemodynamic responses within IFG, and BOLD signal changes paralleled the extent of this manipulation as long as intelligibility of the verbal utterances was preserved [23]. Similarly, sighted subjects showed reduced IFG activation in the present investigation while listening to ultra-fast speech. A further fMRI experiment revealed learning to understand time-compressed speech to be associated with increased activation of left and right auditory association cortices as well as left ventral premotor cortex, suggesting speech perception to involve the integration of multi-modal data sets, mapping acoustic patterns onto articulatory motor plans [74]. At very high syllable rates, sighted subjects, obviously, do not recruit the visual system in order to enhance speech comprehension. Furthermore, invasive electrophysiological measurements during application of time-compressed speech revealed the speech envelope – up to frequencies of 15 Hz – to be well-represented at the level of auditory cortex, suggesting that the time resolution of primary auditory cortex is not the limiting factor for ultra-fast speech comprehension [75]. Similarly, our group found significant MEG phase locking to envelope features of ultra-fast verbal utterances (16 syl/s) [49,76]. In this latter study, blind individuals showed an additional phase-locked component bound to right visual cortex – absent in sighted subjects. Although, principally, primary auditory cortex should be able to track the speech envelope, this extracted information might not suffice to trigger phonological processes during lexical encoding at the level of the working memory.

A recent fMRI study – delineating the “bottleneck” of time-compressed speech processing – found higher stages of language processing associated with “buffer regions” within left ventrolateral frontal cortex/anterior insula, precentral gyrus and mesio-frontal areas to represent the limiting factor of spoken language comprehension [77]. Our data suggest that the visual cortex must also be considered an essential prerequisite to enhanced speech encoding at high syllable rates. Altogether, sighted subjects appear unable – or at least not to “attempt” – to recruit the central-visual system in order to speed up comprehension of spoken language. Occipital cortex, indeed, responds to auditory stimulation, given the negative values of percent signal change, but appears rather to be “actively suppressed” during attempts to understand ultra-fast speech. Against this background, the bottleneck within the frontal language network referred to should represent the upper limit of spoken language understanding. Blind subjects might be able to circumvent these constraints, based upon the recruitment of an additional timing mechanism bound to interactions between pulvinar, auditory/visual cortex, and SMA.

### Limitations of the study

The present study did not find onset of vision loss to pose major constraints upon ultra-fast speech perception capabilities. However, larger well-documented subject groups are required to further corroborate these findings and to identify other clinical factors, such as disease duration, with an eventual impact upon the recruitment of central-visual structures during auditory language comprehension. Furthermore, intra-individual long-term studies are needed to track the time-course of the cerebral reorganization processes associated with the acquisition of ultra-fast speech perception skills and to determine in how far vision loss represents a necessary pre-condition for this capacity. In order to further delineate any differential task-dependent cross-modal reorganization patterns in subjects with early and late vision loss, a larger sample of early-blind individuals has to be recruited.

## Conclusions

Besides the more or less expected responses of perisylvian “language zones”, hemodynamic activation of right-hemispheric V1, contralateral FG, and bilateral Pv was found to covary with ultra-fast speech comprehension capabilities. (i) FG, an area known to be engaged in phonological processing, appears to contribute to the extraction/representation of segmental information of the acoustic speech signal and, thus, to “extend” the left perisylvian network of spoken language processing. (ii) By contrast, right V1 might support, concomitant with Pv and auditory cortex, the encoding of early suprasegmental aspects of the speech signal, feeding, e.g., trigger signals derived from these data structures via left SMA into the perisylvian speech/language network that help to facilitate the consolidation of linguistic information. This model assumes that extant structures and pathways of the central-visual system, disconnected from modality-specific afferent input, are able to enhance behavioral performance within other domains via cross-modal pathways.

## Competing interests

There are no conflicts of interest for any author.

## Authors’ contribution

HA, IH, and SD delineated the rationale and developed the design of the study. IH and SD were engaged in data collection and development of analyses methods. SD performed the behavioral and fMRI data analyses, and drafted the first version of the paper. All authors contributed to the final version of the manuscript and approved its content.

## Supplementary Material

Additional file 1 An example of ultra-fast speech: “Die Billigöfen aus dem Baumarkt scheiden bei einer Umweltbewertung deutlich schlechter ab als etwa Holzpelletöfen, die mit einer steuerbaren Verbrennungsluftregelung und anderen Mechanismen ausgestattet sind.“ Click here for file

Additional file 2 An example of moderately fast speech: “Hinzu kommt eine mangelnde Kompetenz vieler Hausärzte und schlechte Versorgungsstrukturen.“ Click here for file

Additional file 3 **An example of reversed ultra-fast speech (see Additional file **1**).** Click here for file

Additional file 4 **An example of reversed moderately fast speech (see Additional file **2**).** Click here for file

Additional file 5 An example from the repetition task concerning ultra-fast speech comprehension in a blind listener with more than 90% correctly reproduced words: “Öfen, die die Grenzwerte einhalten, kosten zwischen 500 und 700.” Click here for file

Additional file 6 **Coordinates of the whole-head analysis on the impact of speaking rate on hemodynamic brain activation: SPM *T*-contrasts of each condition (ultra-fast, moderately fast/forward, reversed) versus baseline and vice versa. Displayed are the responses exceeding a threshold of *p *< .001 (uncorrected) at a voxel level and *p *< .05 (corrected) at a cluster level, including an extent threshold of *k *(contiguous voxels).** Click here for file

Additional file 7 **Whole-head between-group analysis (blind versus sighted), including each of the various experimental conditions (versus baseline). **Displayed are the responses exceeding a threshold of *p *< .001 (uncorrected) at a voxel level. Click here for file

Additional file 8 **Coordinates of the whole-head between-group analysis (blind versus sighted, experimental conditions versus baseline). **Hemodynamic responses exceeding a threshold of *p *< .001 (uncorrected) at a voxel level and *p* < .05 (corrected, *k *= 68) at a cluster level are displayed, in addition, the activation of further interesting regions, though non-significant at the level of the corrected threshold. Click here for file

Additional file 9 **Coordinates of the whole-head between-group analysis (blind versus sighted), comparing late- and early-blind individuals versus sighted controls each (SPM *T*-contrasts of the condition “all versus baseline”), displayed are the hemodynamic responses exceeding a threshold of *p *< .005 (uncorrected) at a voxel level and *p *< .05 (corrected) at a cluster level as well as the activation of some further relevant regions, though non-significant at the level of the corrected threshold (*k *≥ 15).** Click here for file

Additional file 10 **Whole-head covariance analysis across (i) all subjects, (ii) early-blind individuals removed, and (iii) exclusively late-blind participants (see below).** SPM *T*-contrasts identified the correlation between BOLD responses and ultra-fast speech comprehension capabilities, based upon the condition “ultra-fast versus baseline”. Upper row: 3 early-blind (EB) + 11 late-blind (LB) + 12 sighted subjects (SI), threshold *p *< .001 (uncorrected) at a voxel level; middle row: 11 LB + 12 SI, threshold *p *< .001 (uncorrected) at a voxel level; lower row: 11 LB, threshold *p *< .05 (uncorrected) at a voxel level. Click here for file

Additional file 11 **Coordinates of the whole-head covariance analysis across (i) all subjects, (ii) early-blind individuals removed, and (iii) exclusively late-blind participants (see Additional file **10**). **SPM *T*-contrasts identified the correlation between BOLD responses and ultra-fast speech comprehension capabilities, based upon the condition “ultra-fast versus baseline”. Displayed are the hemodynamic responses exceeding a threshold of *p *< .005 (uncorrected) at a voxel level and *p *< .05 (corrected) at a cluster level, in addition, activation of some further regions, though non-significant at the level of the corrected threshold (across all subjects: *k *≥ 70; LB + SI: *k* ≥ 10), is shown. Click here for file

Additional file 12 **Region-of-interest (ROI) analyses exemplified for three areas, i.e., right-hemispheric primary visual cortex (V1), left-hemispheric fusiform gyrus (FG), and left-hemispheric inferior frontal gyrus (IFG). **Displayed is the strength of hemodynamic responses (% signal change) is displayed within the respective ROIs during each of the following conditions (versus baseline): uf = ultra-fast speech, mf = moderately fast speech, rev-mf = reversed moderately fast speech, rev-uf = reversed ultra-fast speech (error bars = standard error of the mean across subjects; 14 blind, 12 sighted individuals; asterisk = significant (one-sample T-test) Bold responses). Click here for file

Additional file 13 **Correlations between signal change (%) and behavioral performance, onset and duration of blindness, taking into account moderately fast and ultra-fast speech materials. **Values indicate correlations (two-tailed Pearson test) between the moderately fast (mf) or ultra-fast (uf) speech condition (versus baseline) and behavioral performance, onset and duration of vision loss. Upper values: correlation coefficient *r*; lower values in parentheses: significance *p*; bold numbers: significant results at the threshold *p *< .05 Click here for file
