# Supplementary material for: Ultra-fast speech comprehension in blind subjects engages primary visual cortex, fusiform gyrus, and pulvinar – a functional magnetic resonance imaging (fMRI) study
Source: BMC Neurosci. 2013 Jul 23;14:74. doi: 10.1186/1471-2202-14-74 (PMC3847124; doi:10.1186/1471-2202-14-74)
Supplement: Additional file 6 — Coordinates of the whole-head analysis on the impact of speaking rate on hemodynamic brain activation: SPM T-contrasts of each condition (ultra-fast, moderately fast/forward, reversed) versus baseline and vice versa. Displayed are the responses exceeding a threshold of p < .001 (uncorrected) at a voxel level and p < .05 (corrected) at a cluster level, including an extent threshold of k (contiguous voxels). [file 1471-2202-14-74-S6.docx]

| **Additional file 6** Coordinates of the whole-head analysis on the impact of speaking rate on hemodynamic brain activation: SPM *T*-contrasts of each condition (ultra-fast, moderately fast / forward, reversed) versus baseline and vice versa. Displayed are the responses exceeding a threshold of *p* < .001 (uncorrected) at a voxel level and *p* < .05 (corrected) at a cluster level, including an extent threshold of *k* (contiguous voxels). | | | | | | | | | | | | | | | | | | | | | | | | | | | | | | | | |  |  |
| --- | --- | --- | --- | --- | --- | --- | --- | --- | --- | --- | --- | --- | --- | --- | --- | --- | --- | --- | --- | --- | --- | --- | --- | --- | --- | --- | --- | --- | --- | --- | --- | --- | --- | --- |
|  |  | | | | | | | | | | | | | | | |  | |  | | | | | | | | | | | | | |  |  |
|  | 14 blind subjects  14 blind subjects | | | | | | | | | | | | | | | |  | | 12 sighted subjects | | | | | | | | | | | | | |  |  |
| Anatomical region | Side | | Cluster size (voxel) | | | | MNI coordinates | | | | | | | | T value | |  | | Cluster size (voxel) | | | MNI coordinates | | | | | | | | | T value | | |  |
|  |  | |  |  |  |  |  | | | | | | | |  | |  | |  |  |  |  | | | | | | | | |  | | |  |
|  |  | |  | | | | x | | y | | | z | | |  | |  | |  |  | | x | | y | | | | z | | |  | | |  |
| **1. moderately fast versus baseline (k = 59)** | | | | | | | | | | | | | | | | | | | | | | | | | | | | | | | | |  |  |
| **1. Moderately fast speech versus baseline (k = 59)** | | | | | | | | | | | | | | | | | | | | | | | | | | | | | | | | |  |  |
| Superior temporal gyrus | right | 2335 | | | 66 | | | -27 | | | 3 | | | 13.68 | | |  | | 1454 | | 63 | | | | -21 | | 6 | | | 13.95 | | |  |  |
| Heschl's gyrus, TE1.0 | right |  | | | 51 | | | -12 | | | 6 | | | 12.70 | | |  | |  | | 51 | | | | -12 | | 3 | | | 13.45 | | |  |  |
| Heschl's gyrus, TE1.1 | left | 2248 | | | -39 | | | -27 | | | 9 | | | 12.76 | | |  | | 1622 | | -39 | | | | -30 | | 12 | | | 12.34 | | |  |  |
| Inferior frontal gyrus | left |  | | | -51 | | | 18 | | | 15 | | | 4.95 | | |  | | 61 | | -51 | | | | 18 | | 15 | | | 5.00 | | |  |  |
| Precentral gyrus | right | 194 | | | 54 | | | 3 | | | 48 | | | 5.10 | | |  | |  | |  | | | |  | |  | | |  | | |  |  |
| Cerebellum VIII | left | 168 | | | -36 | | | -57 | | | -48 | | | 6.80 | | |  | |  | |  | | | |  | |  | | |  | | |  |  |
| Precentral gyrus, BA6 | left | 134 | | | -51 | | | -6 | | | 51 | | | 5.15 | | |  | |  | |  | | | |  | |  | | |  | | |  |  |
| BA 17 | right | 130 | | | 18 | | | -102 | | | 12 | | | 5.20 | | |  | |  | |  | | | |  | |  | | |  | | |  |  |
| Thalamus | left | 105 | | | -12 | | | -30 | | | -3 | | | 6.28 | | |  | |  | |  | | | |  | |  | | |  | | |  |  |
| Supplementary motor area | left | 80 | | | 0 | | | 9 | | | 69 | | | 4.56 | | |  | | 84 | | -3 | | | | 3 | | 63 | | | 5.80 | | |  |  |
| Cerebellum Crus2 | right |  | | |  | | |  | | |  | | |  | | |  | | 62 | | 15 | | | | -78 | | -39 | | | 5.99 | | |  |  |
|  | | | | | | | | | | | | | | | | | | | | | | | | | | | | | |  | | |  |  |
| **1.1 Baseline versus moderately fast speech**  **1.1 baseline versus moderately fast speech** | | | | | | | | | | | | | | | | | | | | | | | | | | | | | |  | | |  |  |
| Superior medial gyrus | right | | | 745 | | 3 | | | | 51 | | | 6 | | | 8.76 | |  | 658 | | | | 3 | | | 54 | | | 6 | | | 7.20 | | |
| Precuneus | right | | | 529 | | 9 | | | | -54 | | | 48 | | | 6.14 | |  | 198 | | | | 9 | | | -57 | | | 54 | | | 4.58 | | |
| Middle frontal gyrus | right | | | 237 | | 27 | | | | 33 | | | 39 | | | 5.91 | |  |  | | | |  | | |  | | |  | | |  | | |
| Middle occipital gyrus | right | | | 189 | | 42 | | | | -66 | | | 27 | | | 7.54 | |  | 156 | | | | 48 | | | -66 | | | 27 | | | 5.34 | | |
| Middle occipital gyrus | left | | | 105 | | -39 | | | | -84 | | | 36 | | | 5.98 | |  | 78 | | | | -36 | | | -84 | | | 27 | | | 5.82 | | |
| Cuneus | left | | | 100 | | -15 | | | | -57 | | | 18 | | | 5.96 | |  |  | | | |  | | |  | | |  | | |  | | |
| Precuneus | right | | |  | |  | | | |  | | |  | | |  | |  | 91 | | | | 6 | | | -64 | | | 27 | | | 4.92 | | |
| Middle frontal gyrus | left | | | 83 | | -27 | | | | 36 | | | 45 | | | 6.02 | |  | 230 | | | | -27 | | | 36 | | | 45 | | | 6.19 | | |
| Superior frontal gyrus | right | | |  | |  | | | |  | | |  | | |  | |  | 336 | | | | 24 | | | 45 | | | 42 | | | 7.79 | | |
| Middle cingulate cortex | left | | |  | |  | | | |  | | |  | | |  | |  | 60 | | | | -3 | | | -36 | | | 48 | | | 4.94 | | |
|  | | | | | | | | | | | | | | | | | | | | | | | | | | | | | |  | | |  |  |
| **2. Ultra-fast speech versus baseline (k = 68)** | | | | | | | | | | | | | | | | | | | | | | | | | | | | | |  | | |  |  |
| Heschl's gyrus, TE1.1 | Left | | | 1812 | | -39 | | | | -27 | | | 9 | | | 9.37 | |  | 1156 | | | | -39 | | | -33 | | | 12 | | | 12.53 | | |
| Inferior frontal gyrus | left | | |  | | -48 | | | | 21 | | | 15 | | | 6.45 | |  |  | | | |  | | |  | | |  | | |  | | |
| Superior temporal gyrus | right | | | 1402 | | 60 | | | | -15 | | | 9 | | | 10.34 | |  |  | | | |  | | |  | | |  | | |  | | |
| Heschl's gyrus, TE1.0 | right | | |  | | 51 | | | | -12 | | | 3 | | | 10.07 | |  | 1192 | | | | 51 | | | -12 | | | 3 | | | 12.98 | | |
| Cerebellum VIII | right | | | 363 | | 33 | | | | -66 | | | -48 | | | 5.94 | |  |  | | | |  | | |  | | |  | | |  | | |
| Fusiform gyrus | left | | | 262 | | -42 | | | | -54 | | | -21 | | | 4.09 | |  |  | | | |  | | |  | | |  | | |  | | |
| Supplementary motor area | left | | | 191 | | -6 | | | | 6 | | | 60 | | | 4.79 | |  |  | | | |  | | |  | | |  | | |  | | |
| Cuneus, BA17 / 18 | right | | | 177 | | 15 | | | | -102 | | | 6 | | | 5.65 | |  |  | | | |  | | |  | | |  | | |  | | |
| Precentral gyrus | left | | | 160 | | -42 | | | | 0 | | | 39 | | | 4.92 | |  |  | | | |  | | |  | | |  | | |  | | |
| Cerebellum VIII | left | | | 153 | | -27 | | | | -66 | | | -51 | | | 5.36 | |  |  | | | |  | | |  | | |  | | |  | | |
| Inferior frontal gyrus | right | | | 122 | | 45 | | | | 15 | | | 27 | | | 3.99 | |  |  | | | |  | | |  | | |  | | |  | | |
|  | | | | | | | | | | | | | | | | | | | | | | | | | | | | | |  | | |  |  |
| **2.1 Baseline versus ultra-fast speech**  **2.1 baseline versus ultra-fast speech** | | | | | | | | | | | | | | | | | | | | | | | | | | | | | |  | | |  |  |
| Superior medial gyrus | right | | | 611 | | 3 | | | | 51 | | | 6 | | | 6.19 | |  |  | | | |  | | |  | | |  | | |  | | |
| Middle occipital gyrus | right | | | 283 | | 42 | | | | -69 | | | 27 | | | 6.15 | |  |  | | | |  | | |  | | |  | | |  | | |
| Precuneus | right | | | 279 | | 9 | | | | -54 | | | 48 | | | 4.78 | |  |  | | | |  | | |  | | |  | | |  | | |
| Precuneus | left | | |  | |  | | | |  | | |  | | |  | |  | 154 | | | | -9 | | | -75 | | | 51 | | | 4.68 | | |
| Superior frontal gyrus | right | | | 194 | | 24 | | | | 39 | | | 45 | | | 4.51 | |  |  | | | |  | | |  | | |  | | |  | | |
| Parahippocampal gyrus | left | | |  | |  | | | |  | | |  | | |  | |  | 104 | | | | -27 | | | -30 | | | -15 | | | 5.86 | | |
| Middle temporal gyrus | right | | |  | |  | | | |  | | |  | | |  | |  | 83 | | | | 60 | | | -63 | | | 18 | | | 4.30 | | |
|  | | | | | | | | | | | | | | | | | | | | | | | | | | | | | |  | | |  |  |
| **3. Reversed moderately fast speech versus baseline (k = 66)** | | | | | | | | | | | | | | | | | | | | | | | | | | | | | |  | | |  |  |
| Heschl's gyrus, TE1.0 | right | | | 1814 | | 51 | | | | -12 | | | 3 | | | 12.38 | |  | 1203 | | | | 51 | | | -12 | | | 3 | | | 11.82 | | |
| Heschl's gyrus, TE1.1 | left | | | 1668 | | -39 | | | | -30 | | | 12 | | | 11.41 | |  | 1267 | | | | -39 | | | -30 | | | 12 | | | 12.27 | | |
| Cerebellum VIII | left | | | 210 | | -33 | | | | -60 | | | -48 | | | 6.40 | |  | 168 | | | | -33 | | | -63 | | | -48 | | | 5.83 | | |
| Cerebellum VIII | right | | | 154 | | 27 | | | | -63 | | | -48 | | | 5.48 | |  |  | | | |  | | |  | | |  | | |  | | |
| Supplementary motor area | right | | | 120 | | 3 | | | | 12 | | | 60 | | | 4.31 | |  |  | | | |  | | |  | | |  | | |  | | |
| Inferior frontal gyrus | right | | | 116 | | 51 | | | | 12 | | | 33 | | | 4.70 | |  |  | | | |  | | |  | | |  | | |  | | |
| Fusiform gyrus | left | | | 109 | | -42 | | | | -57 | | | -21 | | | 4.11 | |  |  | | | |  | | |  | | |  | | |  | | |
| Precentral gyrus | left | | | 90 | | -60 | | | | 6 | | | 36 | | | 4.19 | |  |  | | | |  | | |  | | |  | | |  | | |
|  | | | | | | | | | | | | | | | | | | | | | | | | | | | | | |  | | |  |  |
| **3.1 Baseline versus reversed moderately fast speech**  **3.1 baseline versus reversed moderately fast speech** | | | | | | | | | | | | | | | | | | | | | | | | | | | | | |  | | |  |  |
| Anterior cingulate cortex | right | | |  | |  | | | |  | | |  | | |  | |  | 419 | | | | 9 | | | 36 | | | -6 | | | 4.65 | | |
| Precuneus | left | | |  | |  | | | |  | | |  | | |  | |  | 191 | | | | 3 | | | -60 | | | 27 | | | 5.03 | | |
|  | | | | | | | | | | | | | | | | | | | | | | | | | | | | | |  | | |  |  |
| **4. Reversed ultra-fast speech versus baseline (k = 58)** | | | | | | | | | | | | | | | | | | | | | | | | | | | | | |  | | |  |  |
| Heschl's gyrus, TE1.0 | right | | | 1320 | | 51 | | | | -12 | | | 3 | | | 12.10 | |  | 1570 | | | | 51 | | | -12 | | | 3 | | | 13.93 | | |
| Heschl's gyrus, TE1.1 | left | | | 1266 | | -39 | | | | -27 | | | 9 | | | 11.89 | |  | 1379 | | | | -39 | | | -27 | | | 9 | | | 13.53 | | |
|  | | | | | | | | | | | | | | | | | | | | | | | | | | | | | |  | | |  |  |
| **4.1 Baseline versus reversed ultra-fast speech**  **4.1 baseline versus reversed ultra-fast speech** | | | | | | | | | | | | | | | | | | | | | | | | | | | | | |  | | |  |  |
| Middle cingulate cortex | left | | | 1165 | | -9 | | | | -39 | | | 45 | | | 6.36 | |  |  | | | |  | | |  | | |  | | |  | | |
| Superior medial gyrus | right | | | 353 | | 6 | | | | 51 | | | 3 | | | 4.86 | |  |  | | | |  | | |  | | |  | | |  | | |
| Middle occipital gyrus | right | | | 321 | | 42 | | | | -66 | | | 24 | | | 9.85 | |  |  | | | |  | | |  | | |  | | |  | | |
| Parahippocampal gyrus | right | | | 155 | | 30 | | | | -30 | | | -18 | | | 5.87 | |  |  | | | |  | | |  | | |  | | |  | | |
| Superior frontal gyrus | right | | | 143 | | 24 | | | | 6 | | | 57 | | | 5.27 | |  |  | | | |  | | |  | | |  | | |  | | |
| Linual gyrus | left | | | 116 | | -24 | | | | -45 | | | -6 | | | 4.97 | |  |  | | | |  | | |  | | |  | | |  | | |
|  | | | | | | | | | | | | | | | | | | | | | | | | | | | | | | | | |  |  |
| Abbreviations: BA, Brodman area | | | | | | | | | | | | | | | | | | | | | | | | | | | | | | | | |  |  |
