# Supplementary figures and images for: Ultra-fast speech comprehension in blind subjects engages primary visual cortex, fusiform gyrus, and pulvinar – a functional magnetic resonance imaging (fMRI) study
Source: BMC Neurosci. 2013 Jul 23;14:74. doi: 10.1186/1471-2202-14-74 (PMC3847124; doi:10.1186/1471-2202-14-74)

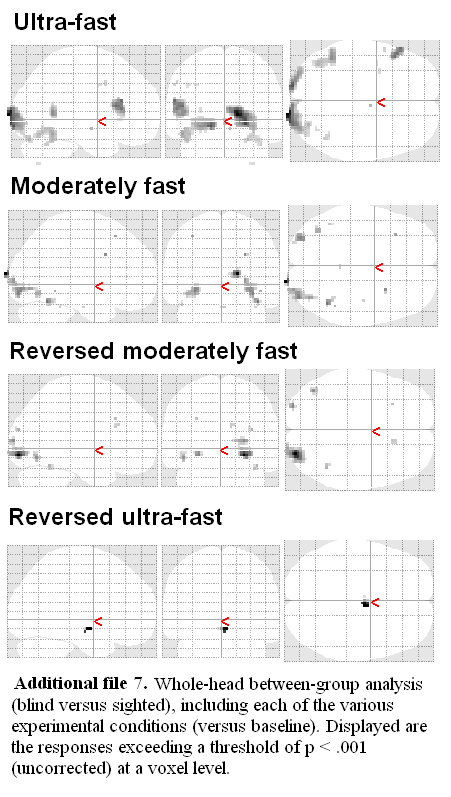

Supplement: Additional file 7 — Whole-head between-group analysis (blind versus sighted), including each of the various experimental conditions (versus baseline). Displayed are the responses exceeding a threshold of p < .001 (uncorrected) at a voxel level. [file 1471-2202-14-74-S7.tiff]

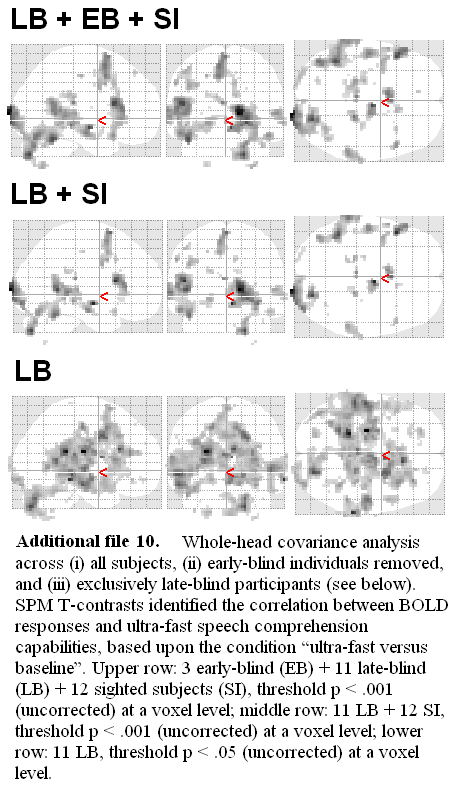

Supplement: Additional file 10 — Whole-head covariance analysis across (i) all subjects, (ii) early-blind individuals removed, and (iii) exclusively late-blind participants (see below). SPM T-contrasts identified the correlation between BOLD responses and ultra-fast speech comprehension capabilities, based upon the condition “ultra-fast versus baseline”. Upper row: 3 early-blind (EB) + 11 late-blind (LB) + 12 sighted subjects (SI), threshold p < .001 (uncorrected) at a voxel level; middle row: 11 LB + 12 SI, threshold p < .001 (uncorrected) at a voxel level; lower row: 11 LB, threshold p < .05 (uncorrected) at a voxel level. [file 1471-2202-14-74-S10.tiff]

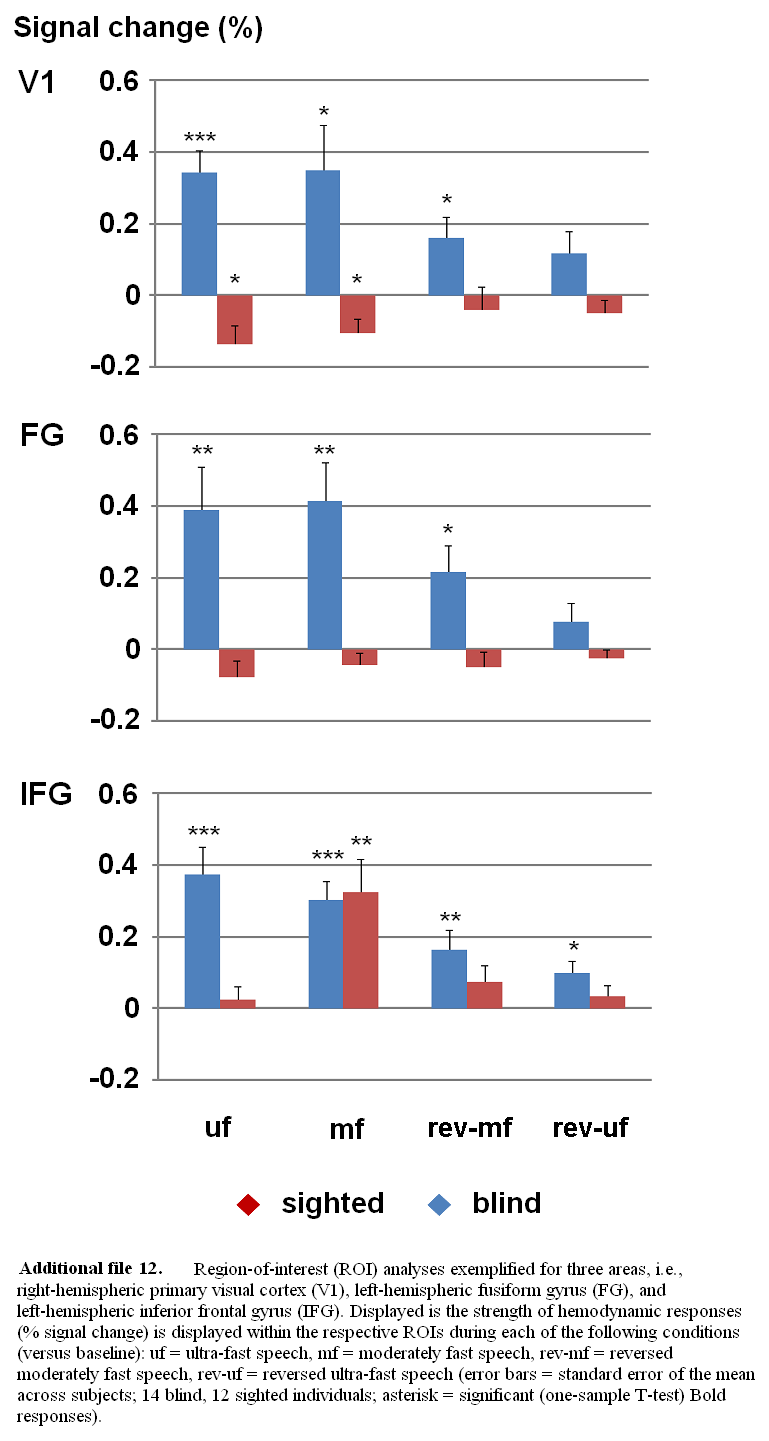

Supplement: Additional file 12 — Region-of-interest (ROI) analyses exemplified for three areas, i.e., right-hemispheric primary visual cortex (V1), left-hemispheric fusiform gyrus (FG), and left-hemispheric inferior frontal gyrus (IFG). Displayed is the strength of hemodynamic responses (% signal change) is displayed within the respective ROIs during each of the following conditions (versus baseline): uf = ultra-fast speech, mf = moderately fast speech, rev-mf = reversed moderately fast speech, rev-uf = reversed ultra-fast speech (error bars = standard error of the mean across subjects; 14 blind, 12 sighted individuals; asterisk = significant (one-sample T-test) Bold responses). [file 1471-2202-14-74-S12.tiff]
