# Supplementary material for: Ultra-fast speech comprehension in blind subjects engages primary visual cortex, fusiform gyrus, and pulvinar – a functional magnetic resonance imaging (fMRI) study
Source: BMC Neurosci. 2013 Jul 23;14:74. doi: 10.1186/1471-2202-14-74 (PMC3847124; doi:10.1186/1471-2202-14-74)
Supplement: Additional file 8 — Coordinates of the whole-head between-group analysis (blind versus sighted, experimental conditions versus baseline). Hemodynamic responses exceeding a threshold of p < .001 (uncorrected) at a voxel level and p < .05 (corrected, k = 68) at a cluster level are displayed, in addition, the activation of further interesting regions, though non-significant at the level of the corrected threshold. [file 1471-2202-14-74-S8.docx]

| **Additional file 8** Coordinates of the whole-head between-group analysis (blind versus sighted, experimental conditions versus baseline). Hemodynamic responses exceeding a threshold of *p* < .001 (uncorrected) at a voxel level and *p* < .05 (corrected, *k* = 68) at a cluster level are displayed, in addition, the activation of further interesting regions, though non-significant at the level of the corrected threshold. | | | | | | |
| --- | --- | --- | --- | --- | --- | --- |
|  | | | | | | |
| Anatomical region | Side | Cluster size  (voxel) | MNI coordinate | | | T value |
|  |  |  | x | y | z |  |
|  |  |  |  |  |  |  |
| **a) Ultra-fast speech (cluster with ≥ 10 voxel are displayed)** | | | | | | |
| Cuneus, BA 17, 18 | right | 260 | 15 | -102 | 6 | 6.13 |
| *SP*: Cerebellum | right |  | 27 | -78 | -21 | 3.99 |
| Calcarine gyrus | left | 91 | -15 | -96 | -6 | 3.47 |
| Inferior temporal gyrus | left | 82 | -48 | -51 | -15 | 4.17 |
| *SP*: Fusiform gyrus | left |  | -39 | -66 | -15 | 3.97 |
| Inferior frontal gyrus | left | 78 | -48 | 21 | 15 | 4.79 |
| Middle temporal gyrus | left | 46 | -45 | -51 | 9 | 4.44 |
| Fusiform gyrus | right | 10 | 39 | -54 | -21 | 3.66 |
| **b) Moderately fast speech (cluster with ≥ 2 voxel are displayed)** | | | | | | |
| Inferior occipital gyrus | right | 74 | 36 | -87 | -9 | 3.99 |
| Inferior occipital gyrus | left | 35 | -30 | -84 | -6 | 3.99 |
| Cuneus, BA 17, 18 | right | 15 | 18 | -102 | 12 | 4.72 |
| Fusiform gyrus | right | 14 | 42 | -54 | -18 | 4.05 |
| Inferior occipital gyrus | left | 12 | -39 | -63 | -9 | 3.77 |
| Fusiform gyrus | left | 2 | -54 | -51 | -21 | 3.61 |
| Cerebellar vermis | left | 2 | 3 | -39 | -24 | 3.61 |
| **c) Reversed moderately fast speech (cluster with ≥ 5 voxel are displayed)** | | | | | | |
| Lingual gyrus, BA 17, 18 | right | 99 | 27 | -87 | -6 | 4.95 |
| Inferior occipital gyrus | left | 15 | -42 | -66 | -9 | 3.97 |
| Inferior occipital gyrus | left | 12 | -24 | -90 | -6 | 4.05 |
| Middle occipital gyrus | right | 5 | 36 | -87 | 21 | 3.80 |
| Fusiform gyrus | right | 5 | 42 | -51 | -18 | 3.76 |
| **d) Reversed ultra-fast speech** | | | | | | |
| Capsule interna | right | 9 | 6 | -6 | 9 | 4.78 |
| Abbreviations: BA, Brodman area; MNI, Montreal Neuroscience Institute template; T, height threshold; SP, sub-peak. | | | | | | |
