# Supplementary material for: Ultra-fast speech comprehension in blind subjects engages primary visual cortex, fusiform gyrus, and pulvinar – a functional magnetic resonance imaging (fMRI) study
Source: BMC Neurosci. 2013 Jul 23;14:74. doi: 10.1186/1471-2202-14-74 (PMC3847124; doi:10.1186/1471-2202-14-74)
Supplement: Additional file 9 — Coordinates of the whole-head between-group analysis (blind versus sighted), comparing late- and early-blind individuals versus sighted controls each (SPM T-contrasts of the condition “all versus baseline”), displayed are the hemodynamic responses exceeding a threshold of p < .005 (uncorrected) at a voxel level and p < .05 (corrected) at a cluster level as well as the activation of some further relevant regions, though non-significant at the level of the corrected threshold (k ≥ 15). [file 1471-2202-14-74-S9.docx]

| Additional file 9 Coordinates of the whole-head between-group analysis (blind versus sighted), comparing late- and early-blind individuals versus sighted controls each (SPM *T*-contrasts of the condition “all versus baseline”), displayed are the hemodynamic responses exceeding a threshold of *p* < .005 (uncorrected) at a voxel level and *p* < .05 (corrected) at a cluster level as well as the activation of some further relevant regions, though non-significant at the level of the corrected threshold (*k* ≥ 15). | | | | | | | |
| --- | --- | --- | --- | --- | --- | --- | --- |
|  | | | | | | | |
| Anatomical region | Side | Cluster size  (voxel) | | MNI coordinate | | | T value |
|  |  |  |  | x | y | z |  |
|  |  |  | |  |  |  |  |
| **a) Early and late blind versus sighted** | | | | | | | |
| Cuneus, BA17 / 18 | right | 360 | | 18 | -102 | 9 | 4.89 |
| Inferior occipital gyrus | left | 200 | | -39 | -63 | -9 | 4.08 |
| *SP*: inferior temporal gyrus | left |  | | -45 | -51 | -18 | 3.89 |
| Inferior occipital gyrus | left | 126 | | -24 | -90 | -3 | 3.92 |
| Fusiform gyrus | right | 55 | | 41 | -51 | -18 | 3.79 |
| Middle temporal gyrus | left | 49 | | -45 | -51 | 9 | 3.51 |
| Internal capsule | right | 22 | | 6 | -6 | -9 | 4.60 |
| Precentral gyrus | left | 18 | | -39 | 0 | 36 | 2.96 |
| **b) Only late blind versus sighted** | | | | | | | |
| Cuneus, BA17 / 18 | right | 219 | | 18 | -102 | 12 | 4.45 |
| Inferior occipital gyrus | left | 111 | | -39 | -63 | -9 | 3.79 |
| *SP*: inferior temporal gyrus | left |  | | -48 | -51 | -18 | 3.62 |
| Inferior occipital gyrus | left | 30 | | -24 | -90 | -6 | 3.41 |
| Middle temporal gyrus | left | 20 | | -48 | -51 | 9 | 3.57 |
| Internal capsule | right | 18 | | 6 | -3 | -9 | 4.85 |
| Fusiform gyrus | right | 17 | | 42 | -51 | -18 | 3.26 |
| **c) Only early blind versus sighted** | | | | | | | |
| Middle occipital gyrus | right | | 1186 | 27 | -90 | 3 | 5.06 |
| *SP*: Middle temporal gyrus | right | |  | 45 | -72 | 3 | 4.85 |
| *SP*: Cuneus | right | |  | 12 | -99 | 6 | 4.18 |
| *SP*: Fusiform gyrus | right | |  | 42 | -48 | -21 | 4.15 |
| Middle temporal gyrus | left | | 603 | -42 | -72 | 6 | 4.78 |
| *SP*: Cerebellum | left | |  | -33 | -81 | -21 | 4.52 |
| *SP*: Fusiform gyrus | left | |  | -39 | -57 | -18 | 4.37 |
| Lingual gyrus | left | | 48 | -9 | -72 | -3 | 3.55 |
| Superior occipital gyrus | right | | 38 | 21 | -87 | 24 | 3.66 |
| Cerebellar vermis | right | | 22 | 0 | -48 | -21 | 6.00 |
| Superior frontal gyrus | right | | 17 | 24 | 60 | 9 | 3.90 |
| Abbreviations: BA, Brodman area; MNI, Montreal Neuroscience Institute template; T, height threshold; SP, sub-peak. | | | | | | | |
