# Supplementary material for: Ultra-fast speech comprehension in blind subjects engages primary visual cortex, fusiform gyrus, and pulvinar – a functional magnetic resonance imaging (fMRI) study
Source: BMC Neurosci. 2013 Jul 23;14:74. doi: 10.1186/1471-2202-14-74 (PMC3847124; doi:10.1186/1471-2202-14-74)
Supplement: Additional file 11 — Coordinates of the whole-head covariance analysis across (i) all subjects, (ii) early-blind individuals removed, and (iii) exclusively late-blind participants (see Additional file 10). SPM T-contrasts identified the correlation between BOLD responses and ultra-fast speech comprehension capabilities, based upon the condition “ultra-fast versus baseline”. Displayed are the hemodynamic responses exceeding a threshold of p < .005 (uncorrected) at a voxel level and p < .05 (corrected) at a cluster level, in addition, activation of some further regions, though non-significant at the level of the corrected threshold (across all subjects: k ≥ 70; LB + SI: k ≥ 10), is shown. [file 1471-2202-14-74-S11.docx]

| **Additional file 11** Coordinates of the whole-head covariance analysis across (i) all subjects, (ii) early-blind individuals removed, and (iii) exclusively late-blind participants (see Additional file 10). SPM *T*-contrasts identified the correlation between BOLD responses and ultra-fast speech comprehension capabilities, based upon the condition “ultra-fast versus baseline”. Displayed are the hemodynamic responses exceeding a threshold of *p* < .005 (uncorrected) at a voxel level and *p* < .05 (corrected) at a cluster level, in addition, activation of some further regions, though non-significant at the level of the corrected threshold (across all subjects: *k* ≥ 70; LB + SI: *k* ≥ 10), is shown. | | | | | | |
| --- | --- | --- | --- | --- | --- | --- |
|  | | | | | | |
| Anatomical region | Side | Cluster size  (voxel) | MNI coordinate | | | T value |
|  |  |  | x | y | z |  |
|  |  |  |  |  |  |  |
| **a) Early-blind, late-blind, and sighted** | | | | | | |
| Fusiform gyrus | left | 672 | -42 | -51 | -21 | 6.07 |
| *SP*: Middle temporal gyrus | left |  | -45 | -48 | 6 | 5.20 |
| *SP*: Inferior occipital gyrus | left |  | -24 | -93 | -6 | 4.19 |
| Cuneus, BA 17, 18 | right | 464 | 15 | -102 | 6 | 7.61 |
| *SP*: Cerebellum | right |  | 21 | -78 | -39 | 6.11 |
| Inferior frontal gyrus | left | 211 | -48 | 18 | 18 | 6.15 |
| Supplementary motor area | left | 184 | -6 | 9 | 60 | 5.42 |
| Precentral gyrus | left | 108 | -42 | 0 | 36 | 4.60 |
| Pulvinar | right | 94 | 18 | -30 | 6 | 5.45 |
| Pulvinar | left | 81 | -18 | -30 | 3 | 4.69 |
| Middle temporal gyrus | right | 71 | 57 | -36 | 6 | 4.84 |
| **b) Late-blind and sighted** | | | | | | |
| Middle temporal gyrus | left | 346 | -45 | -48 | 6 | 5.88 |
| *SP*: Fusiform gyrus | left |  | -42 | -51 | -21 | 5.32 |
| Cuneus, BA 17, 18 | right | 343 | 15 | -102 | 6 | 7.31 |
| *SP*: Cerebellum | right |  | 21 | -78 | -39 | 5.95 |
| Inferior frontal gyrus | left | 120 | -48 | 18 | 15 | 6.41 |
| Supplementary motor area | left | 80 | -6 | 9 | 60 | 4.86 |
| Middle temporal gyrus | left | 55 | -54 | -15 | -15 | 4.98 |
| Middle temporal gyrus | right | 53 | 57 | -36 | 6 | 4.50 |
| Precentral gyrus | left | 30 | -42 | 3 | 33 | 4.46 |
| Pulvinar | right | 13 | 18 | -30 | 6 | 4.80 |
| **c) Only late-blind** | | | | | | |
| Middle temporal gyrus | left | 3440 | -54 | -18 | -15 | 4.78 |
| *SP*: Pulvinar | right |  | 18 | -30 | 6 | 3.90 |
| *SP*: Supplementary motor area | left |  | -6 | 9 | 60 | 2.96 |
| *SP*: Fusiform gyrus | left |  | -42 | -51 | -24 | 2.89 |
|  |  |  |  |  |  |  |
|  |  |  |  |  |  |  |
| *SP*: Precentral gyrus | left |  | -42 | 0 | 33 | 2.31 |
| *SP*: Pulvinar | left |  | -18 | -30 | 3 | 2.68 |
| Abbreviations: BA, Brodman area; MNI, Montreal Neuroscience Institute template; T, height threshold; SP, sub-peak. | | | | | | |
