# Supplementary material for: Ultra-fast speech comprehension in blind subjects engages primary visual cortex, fusiform gyrus, and pulvinar – a functional magnetic resonance imaging (fMRI) study
Source: BMC Neurosci. 2013 Jul 23;14:74. doi: 10.1186/1471-2202-14-74 (PMC3847124; doi:10.1186/1471-2202-14-74)
Supplement: Additional file 13 — Correlations between signal change (%) and behavioral performance, onset and duration of blindness, taking into account moderately fast and ultra-fast speech materials. Values indicate correlations (two-tailed Pearson test) between the moderately fast (mf) or ultra-fast (uf) speech condition (versus baseline) and behavioral performance, onset and duration of vision loss. Upper values: correlation coefficient r; lower values in parentheses: significance p; bold numbers: significant results at the threshold p < .05 [file 1471-2202-14-74-S13.docx]

| **Additional file 13** Correlations between signal change (%) and behavioral performance, onset and duration of blindness, taking into account moderately fast and ultra-fast speech materials. Values indicate correlations (two-tailed Pearson test) between the moderately fast (mf) or ultra-fast (uf) speech condition (versus baseline) and behavioral performance, onset and duration of vision loss. Upper values: correlation coefficient *r*; lower values in parentheses: significance *p*; bold numbers: significant results at the threshold *p* < .05. | | | | | | | | | | | | | | | | | | | | |
| --- | --- | --- | --- | --- | --- | --- | --- | --- | --- | --- | --- | --- | --- | --- | --- | --- | --- | --- | --- | --- |
|  | | | | | | | | | | | | | | | | | | | | |
|  | V1 | | FG | | IFG | | SMA | | PrCG | | aSTS | | pSTS (LH) | | pSTS (RH) | | Pv (LH) | | Pv (RH) | |
|  | mf | uf | mf | uf | mf | uf | mf | uf | mf | uf | mf | uf | mf | uf | mf | uf | mf | uf | mf | uf |
|  |  |  |  |  |  |  |  |  |  |  |  |  |  |  |  |  |  |  |  |  |
| Performance (blind) | .046 (.877) | **.533 (<.05)** | .369 (.194) | **.650 (<.05)** | .437 (.119) | **.607 (<.05)** | .368 (.196) | **.712 (<.01)** | -.116 (.694) | **.542 (<.05)** | **.586 (<.05)** | **.831 (<.001)** | -.103 (.725) | **.575 (<.05)** | .144 (.624) | **.682 (<.01)** | **.688 (< .01)** | **.687 (<.01)** | **.720 (<.01)** | **.742 (<.01)** |
| Performance (sighted) | .399 (.198) | .452 (.141) | .029 (.929) | -.205 (.523) | .233 (.465) | .190 (.554) | .098 (.761) | .369 (.238) | .095 (.769) | .100 (.758) | .474 (.119) | .379 (.225) | .563 (.057) | .520 (.083) | .130 (.686) | .269 (.397) | .330 (.295) | -.092 (.777) | .354 (.260) | .150 (.643) |
|  |  |  |  |  |  |  |  |  |  |  |  |  |  |  |  |  |  |  |  |  |
| Onset of blindness | -.435 (.120) | -.298 (.301) | -.422 (.133) | -.243 (.403) | -.181 (.535) | -.307 (.285) | -.217 (.457) | -.307 (.286) | .162 (.581) | -.038 (.899) | -.417 (.138) | -.225 (.439) | -.303 (.292) | -.530 (.051) | -.392 (.166) | -.467 (.093) | -.283 (.327) | -.120 (.683) | -.243 (.403) | -.058 (.843) |
|  |  |  |  |  |  |  |  |  |  |  |  |  |  |  |  |  |  |  |  |  |
| Duration of blindness | .441 (.114) | .225 (.439) | .294 (.307) | .413 (.142) | -.081 (.783) | .193 (.508) | -.274 (.344) | .015 (.958) | -.201 (.492) | -.122 (.678) | .283 (.327) | .051 (.864) | -.340 (.234) | .452 (.105) | -.296 (.304) | -.264 (.361) | .081 (.783) | .037 (.900) | -.068 (.818) | .031 (.917) |
|  | | | | | | | | | | | | | | | | | | | | |
| Abbreviations: mf, moderately fast; uf, ultra-fast; V1, primary visual area; FG, fusiform gyrus; IFG, inferior frontal gyrus; aSTS, anterior superior temporal sulcus; pSTS, posterior superior temporal sulcus; PrCG, precentral gyrus; Pv, pulvinar; LH, left-hemisphere; RH, right-hemisphere. | | | | | | | | | | | | | | | | | | | | |
